# Zinc accumulation-induced integrated stress response triggers β-cell identity loss

**DOI:** 10.1038/s41422-026-01222-y

**Published:** 2026-01-28

**Authors:** Qing Ma, Wenjun Xu, Xuan Wang, Haoyu Nie, Yukun Gao, Rui Hu, Zhihao Yang, Xushu Wang, Ta Na, Xiangyi Chen, Zhaoyue Wang, Minglu Xu, Li Shao, Meng Guo, Yanfang Liu, Rongrong Le, Shaorong Gao, Weida Li

**Affiliations:** 1 Institute for Regenerative Medicine, State Key Laboratory of Cardiology and Medical Innovation Center, Shanghai East Hospital, Clinical and Translational Research Center of Shanghai First Maternity & Infant Hospital, Frontier Science Center for Stem Cell Research, Shanghai Key Laboratory of Signaling and Disease Research, School of Life Sciences and Technology, Tongji University, Shanghai, China; 2 Department of Pathology, Changhai Hospital, Navy Medical University, Shanghai, China; 3 Department of VIP Clinic, Shanghai East Hospital, Tongji University School of Medicine, Shanghai, China; 4 Reg-Verse Therapeutics (Shanghai) Co. Ltd., Shanghai, China

**Keywords:** Stem-cell differentiation, Mechanisms of disease

## Abstract

Pancreatic β-cell identity loss is increasingly recognized as a critical pathogenic contributor to β-cell failure in type 2 diabetes (T2D), but the specific mechanism remains to be characterized. In this study, we demonstrate that zinc accumulation contributes to β-cell identity loss during diabetes progression in both human and mouse islets. Using a model of human embryonic stem cell-derived islets (SC-islets), we reveal that accumulated zinc triggers the integrated stress response (ISR), with elevated ATF4 expression in SC-β cells. This, in turn, initiates expression of the α cell-specific transcription factor *ARX*, resulting in the conversion of β cells to α cells, thus forming a zinc-ATF4-ARX regulatory axis. Like primary β cells, SC-β cells also undergo identity loss after transplantation into diabetic animals, which can be prevented by an ISR inhibitor, resulting in improved glycemic control. Furthermore, both genetic depletion and chemical inhibition of zinc accumulation effectively safeguard SC-β cells from identity loss and enhance their efficacy in diabetic animals. Our study thus reveals a pathogenic mechanism in which zinc accumulation induces β-cell identity loss through lineage-tracing approaches and proposes a protective strategy to counteract this process.

## Introduction

Pancreatic β cells are the only cell type that produces insulin, which is crucial for maintaining glycemic homeostasis. Consequently, β-cell loss is a major contributor to the development of type 2 diabetes (T2D).^1–3^ Clinical evidence indicates that the β-cell mass of patients with T2D is an average of 25%–60% lower than that of healthy individuals.^4,5^ Recent studies indicate that this reduction is not due to cell death, but rather to β-cell identity loss^6–10 ^— a process in which β cells lose their mature phenotype, dedifferentiate into progenitor-like cells, or transdifferentiate into other pancreatic endocrine cells under chronic diabetic stress or owing to genetic defects.^11–13^ Loss of β-cell identity has been consistently observed in mouse models,^9^ non-human primates,^14,15^ and patients with T2D,^16–20^ characterized by a reduction in β cells, together with an increase in α cells and bi-hormonal INS^+^GCG^+^ cells. Together, these findings suggest that the loss of pancreatic β-cell identity is a crucial factor in the progression of T2D.

Genetic susceptibility, oxidative stress, and endoplasmic reticulum (ER) stress are reportedly involved in β-cell identity loss.^3,21^ Examples include the loss of key β-cell transcription factors such as FOXO1, which leads to β-cell dedifferentiation, or the loss of PDX1 or NKX6.1, which results in β-cell transdifferentiation.^9,22,23^ In addition, ectopic expression of the α-cell-specific transcription factor ARX in β cells leads to the loss of β-cell phenotypes and the acquisition of α-cell characteristics.^24,25^ Furthermore, the high insulin demand makes β cells especially vulnerable to ER stress,^26^ which together with oxidative stress in diabetes, suppresses β-cell transcription factors (MAFA, PDX1).^27,28^ Notably, impairment of the unfolded protein response (UPR), including XBP1 or IRE1α deficiency, has also been implicated in β-cell transdifferentiation or dedifferentiation.^29,30^ A recent study showed that the mitochondrial integrated stress response (ISR), triggered by dysfunctional mitochondrial quality control, promotes β-cell immaturity.^10^ Despite these findings, current research remains fragmented, relying mainly on gene-specific knockouts and lacking a comprehensive understanding of the etiology of β-cell identity loss in human diabetes. The absence of suitable human models has also hindered mechanistic studies of β-cell identity loss in T2D, a limitation now addressed by human embryonic stem cell (hESC)-derived islets (SC-islets), which are increasingly used to investigate human β-cell development and islet pathology in diabetes.^16,31–33^

Pancreatic β cells contain a large amount of zinc in insulin granules (10–20 mM, ~1000-fold higher than in other cell types).^34^ However, pathological zinc accumulation causes irreversible damage to ATP synthesis and mitochondrial function, ultimately leading to β-cell failure.^35,36^ Moreover, human genetic studies have shown that loss-of-function (LOF) of the islet-specific zinc transporter 8 (ZnT8, encoded by *SLC30A8*) significantly decreases diabetes risk by reducing zinc levels in β cells, thereby enhancing β-cell function.^32,37,38^ These studies highlight the detrimental effects of excessive zinc on β cells. Thus, whether excessive zinc affects β-cell identity is a crucial issue worth exploring.

In this work, we show that accumulated zinc is a crucial pathogenic factor that induces loss of identity in pancreatic β cells of patients with T2D, and we reveal the mechanism by which the zinc-ATF4-ARX axis mediates conversion of β cells to α cells. More importantly, we demonstrate that human primary islets undergo identity loss and zinc accumulation after implantation into diabetic mice, phenomena that are also observed in SC-islets, thereby compromising glycemic recovery. We therefore used genetic and chemical methods to reduce zinc accumulation, effectively protecting SC-β cells from identity loss and enhancing efficacy in diabetic animals. In summary, our findings offer novel insights into protecting β-cell identity.

## Results

### β-cell identity loss is associated with increased zinc transport

**Fig. 1:**
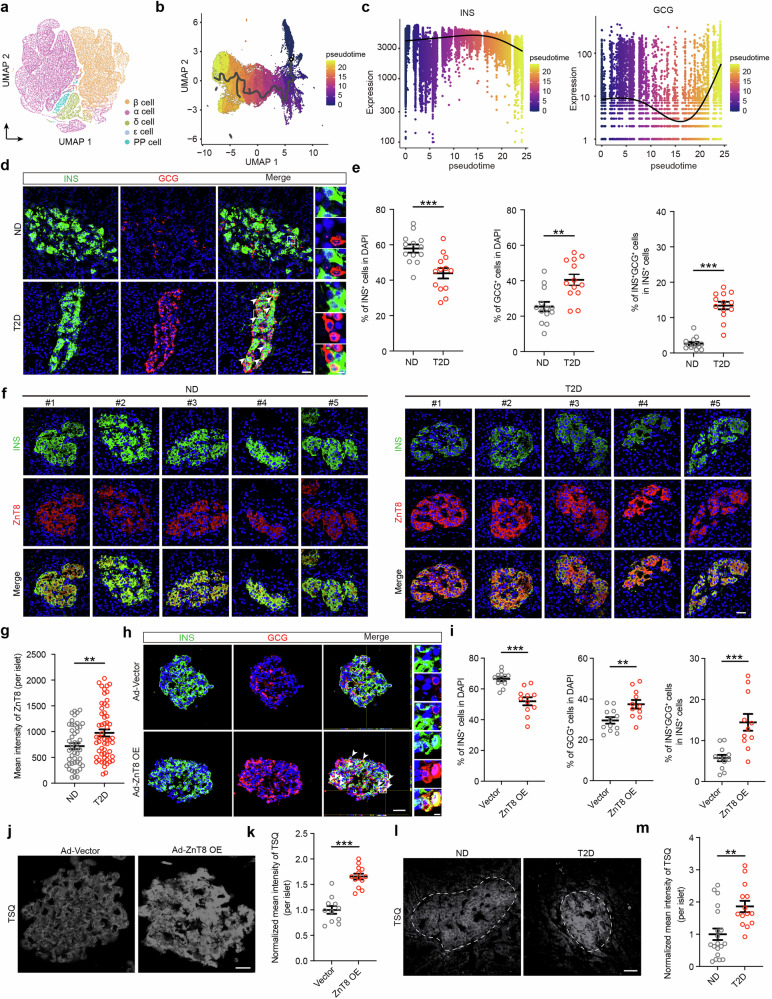
β-cell identity loss correlates with ZnT8 upregulation and subsequent zinc accumulation. **a** UMAP analysis of single cells from human islets of ND individuals (*n* = 23) and patients with T2D (*n* = 15) from the Human Pancreas Analysis Program. Clusters are separated by colors. **b** Single-cell trajectory of human β cells from ND individuals and patients with T2D; cells were ordered by pseudotime along the path from *INS*^high^*GCG*^low^ to *INS*^low^*GCG*^high^ β cells. **c** Changes in the expression of *INS* and *GCG* along pseudotime. **d**, **e** Representative immunostaining for INS (green), GCG (red), and DAPI (blue) (**d**) and quantification (**e**) of the percentages of INS^+^ cells and GCG^+^ cells among the total number of DAPI^+^ cells, as well as the ratio of bi-hormonal INS^+^GCG^+^ cells to total INS^+^ cells, in human islets from ND individuals and patients with T2D. *n* = 13 islets from 3 independent human samples. White arrows indicate bi-hormonal INS^+^GCG^+^ cells. Scale bar (low magnification), 25 μm; scale bar (high magnification), 5 μm. **f**, **g** Representative immunofluorescent images for INS (green) and ZnT8 (red) (**f**) and mean intensity measurements of ZnT8 (**g**) from human islets from ND individuals (*n* = 47 islets from 10 independent human samples) and patients with T2D (*n* = 56 islets from 10 independent human samples). Scale bar, 25 μm. **h**, **i** Representative immunofluorescent Z-stack images (**h**) and quantification (**i**) of the percentages of INS^+^ cells (green) and GCG^+^ cells (red) among the total number of DAPI^+^ cells (blue), as well as the ratio of bi-hormonal INS^+^GCG^+^ cells to total INS^+^ cells, in human islets infected with adenovirus carrying either an empty vector (*n* = 12 islets from 3 independent human samples) or a ZnT8 OE construct (*n* = 11 islets from 3 independent human samples) and cultured in high-glucose medium (33 mM). **j**, **k** White arrows indicate bi-hormonal INS^+^GCG^+^ cells. Scale bar (low magnification), 25 μm; scale bar (high magnification), 5 μm. TSQ staining (**j**) and corresponding normalized mean intensity measurements (**k**) of human primary islets infected with adenovirus carrying either an empty vector (*n* = 11 islets from 3 independent human samples) or a ZnT8 OE construct (*n* = 13 islets from 3 independent human samples). Scale bar, 25 μm. **l**, **m** TSQ staining (**l**) and corresponding normalized mean intensity measurements (**m**) for human islets from ND individuals (*n* = 19 islets from 3 independent human samples) and patients with T2D (*n* = 14 islets from 3 independent human samples). Scale bar, 50 μm. Unpaired two-tailed *t*-test was used to analyze data in this figure. ns not significant; **P < 0.01, ***P < 0.001. Data are presented as mean ± SEM. Individual data points are shown for all bar graphs.

In recent years, several studies have revealed that loss of β-cell identity is a major determinant of β-cell failure.^6,10,15,39^ To characterize the dynamic process of β-cell identity loss, we analyzed single-cell RNA sequencing (scRNA-seq) data from human islets acquired from the Human Pancreas Analysis Program Database.^40^ The dataset included 23 non-diabetic (ND) individuals and 15 patients with T2D (Supplementary information,  Fig. S1a, b and Table S1). Cells from human islets were grouped into five clusters (Fig. 1a) corresponding to five major cell types: β cells (*INS*, *NKX6.1*), α cells (*GCG*, *ARX*), δ cells (*SST*), ε cells (*GHRL*) and pancreatic polypeptide cells (*PPY*) (Supplementary information, Fig. S1c, d). Compositional analysis revealed that the proportion of α cells was higher and the proportion of β cells was lower in islets from patients with T2D than in those from ND individuals (Supplementary information, Fig. S1e).

Using Monocle 3, we reordered pancreatic β cells on the basis of pseudotime analysis (Fig. 1b).^41–47^ We selected the cell cluster with the highest *INS* expression as the starting node (Supplementary information, Fig. S2a) and observed a shift in cell identity from β cells to α cells. At the beginning of the trajectory, β cells exhibited high *INS* expression and low *GCG* expression, whereas at the end of the trajectory, they displayed high *GCG* expression and low *INS* expression (Fig. 1c). In addition, expression of *ARX* (an α cell-specific transcription factor) was upregulated, whereas that of *CHGA* (a marker gene for pancreatic endocrine cells) was unchanged (Supplementary information, Fig. S2b–d). To confirm β-cell identity loss in islets from patients with T2D, we performed immunofluorescence staining of pancreatic sections from patients with T2D and ND individuals using antibodies against INS and GCG. We observed a significant decrease in β cells (INS^+^), accompanied by an increase in α cells (GCG^+^) and bi-hormonal INS^+^GCG^+^ cells, in islets from patients with T2D compared with those from ND individuals (Fig. 1d, e; Supplementary information, Table S2). These compositional changes provide further evidence for β-cell identity loss under diabetic conditions.

ZnT8 also appeared to play a role in β-cell identity loss. Our results revealed that ZnT8 protein levels were substantially higher in islets from patients with T2D (*n* = 10) than in those from ND individuals (*n* = 10) (Fig. 1f, g; Supplementary information, Table S3), consistent with the scRNA-seq analysis (Supplementary information, Fig. S2e, f). Moreover, ZnT8 expression was consistently higher in β cells than in α cells across samples from both ND and T2D individuals (Supplementary information, Fig. S3a, b), and the fold increase in ZnT8 expression was significantly greater in β cells than in α cells from islets of patients with T2D (Supplementary information, Fig. S3a, c), indicating a more pronounced upregulation of ZnT8 in β cells during T2D progression.

To confirm these findings, we used a diabetic mouse model incorporating a lineage-tracing strategy (*RIP-Cre; Rosa26*^*tdTomato*^), which specifically labels the β-cell lineage with tdTomato (Supplementary information, Fig. S4a). After 6 months of a high-fat diet (HFD), the mice developed pronounced overweight phenotypes, characterized by increased body weight, enlarged hepatic lipid droplets, and a higher liver-to-body weight ratio compared with controls fed a normal diet (ND) (Supplementary information, Fig. S4b–e). The emergence of impaired glucose tolerance in HFD-fed mice indicated the successful establishment of the diabetes model (Supplementary information, Fig. S8a).

We next examined whether β cells adopt an α-cell fate following HFD feeding by performing co-immunostaining for tdTomato, INS, and GCG in mouse islets. Co-staining for tdTomato with GCG revealed increased overlap in HFD-fed mice compared with ND-fed controls, suggesting that β cells undergo transdifferentiation into α cells in the islets of HFD-induced diabetic mice. Correspondingly, we observed a reduced percentage of INS^+^ cells and significantly greater proportions of GCG^+^ cells and bi-hormonal INS^+^GCG^+^ cells in the HFD-fed group compared with ND-fed controls (Supplementary information, Fig. S5a, b). Consistent with the upregulation of ZnT8 observed in human diabetic β cells, ZnT8 expression was significantly increased in β cells from HFD-fed mice compared with those from ND-fed controls (Supplementary information, Fig. S5c, d). To determine whether ZnT8 upregulation induces β-cell identity loss, we overexpressed ZnT8 in primary human islets via an adenoviral vector. ZnT8 overexpression in human islets resulted in a significant reduction in β cells, an increase in α cells, and a higher proportion of bi-hormonal INS^+^GCG^+^ cells (Fig. 1h, i). Consistent with these results, ZnT8 was significantly upregulated and showed clear co-localization with INS^+^GCG^+^ cells, with higher ZnT8 intensity in INS^+^GCG^+^ cells than in INS^+^GCG⁻ cells (Supplementary information, Fig. S6a–c). Together, these findings suggest that elevated ZnT8 expression contributes to the loss of β-cell identity.

### Zinc accumulation leads to β-cell identity loss

Given that ZnT8 is the principal zinc transporter in β cells,^48,49^ changes in its expression had a profound effect on intracellular zinc homeostasis (Fig. 1j, k; Supplementary information, Fig. S6d, e). We used TSQ (6-methoxy-8-p-toluenesulfonamido-quinoline) staining to measure intracellular zinc levels^50^ and observed significantly higher zinc levels in islets from patients with T2D than in those from ND controls (Fig. 1l, m; Supplementary information, Fig. S6f and Table S2). Consistent with this finding, higher zinc levels were also observed in tdTomato^+^ cells from HFD-fed mice than in those from ND-fed mice (Supplementary information, Fig. S6g–i). Taken together, these findings indicate that upregulation of ZnT8 leads to β-cell identity loss associated with excessive zinc levels.

**Fig. 2:**
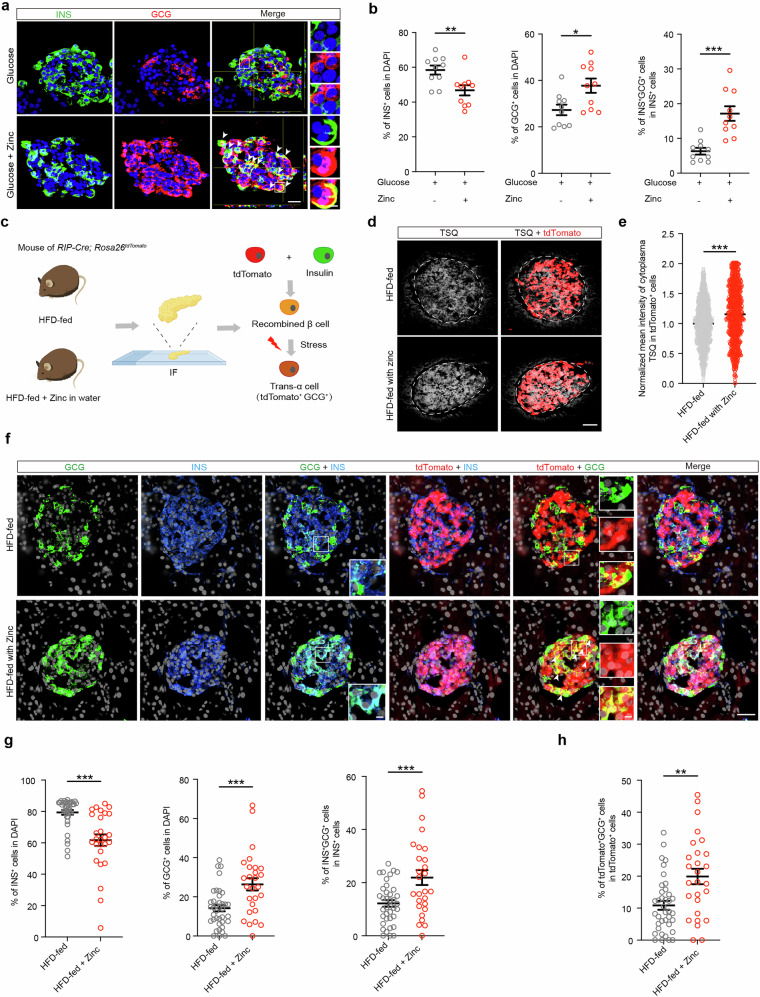
Zinc accumulation leads to β-cell identity loss. **a**, **b** Representative immunofluorescent Z-stack images (**a**) and quantification (**b**) of the percentages of INS^+^ cells (green) and GCG^+^ cells (red) among the total number of DAPI^+^ cells (blue), as well as the ratio of bi-hormonal INS^+^GCG^+^ cells to total INS^+^ cells, in human primary islets with or without excessive zinc (200 μM ZnSO_4_) treatment in a high-glucose (33 mM) environment. *n* = 10 islets from 3 independent human samples. White arrows indicate bi-hormonal INS^+^GCG^+^ cells. Scale bar (low magnification), 25 μm; scale bar (high magnification), 5 μm. **c** Schematic diagram of *RIP-Cre*; *Rosa26*^*tdTomato*^ HFD-fed mice, with or without zinc supplementation, used to trace β-cell fate. **d**, **e** The diagram was created with figdraw.com. TSQ staining (**d**) and normalized mean intensity measurements (**e**) for islets from HFD-fed *RIP-Cre*; *Rosa26*^*tdTomato*^ mice without (*n* = 1208 cells) or with (*n* = 980 cells) zinc supplementation. Scale bar, 50 μm. **f**–**h** Representative immunofluorescence images (**f**) and quantification (**g**, **h**) showing the percentages of INS^+^ cells (blue) and GCG^+^ cells (green) among the total number of DAPI^+^ cells (gray), the proportion of bi-hormonal INS^+^GCG^+^ cells among total INS^+^ cells, and the proportion of tdTomato^+^GCG^+^ cells among total tdTomato^+^ cells in islets from HFD-fed mice (*n* = 38) and HFD-fed mice supplemented with zinc (*n* = 27). Scale bar (low magnification), 25 μm; scale bar (high magnification), 5 μm. Unpaired two-tailed *t*-test was used to analyze data in this figure. ns not significant; *P < 0.05, **P < 0.01, ***P < 0.001. Data are presented as mean ± SEM. Individual data points are shown for all bar graphs.

To further mimic hyperglycemia and zinc accumulation in diabetes, high glucose and excessive zinc were added to the culture medium of human islets. Again, the percentage of β cells was reduced in human islets treated with excessive zinc, whereas the percentages of α cells and bi-hormonal INS^+^GCG^+^ cells were significantly increased (Fig. 2a, b), indicating that excessive zinc leads to β-cell identity loss in a high-glucose environment. Furthermore, treatment with the zinc ionophore zinc pyrithione (ZnPTO, a membrane-permeable compound containing a zinc atom^51^) without high glucose was sufficient to induce β-cell identity loss, manifested as a reduction in β cells and an increase in α cells and bi-hormonal INS^+^GCG^+^ cells in human primary islets (Supplementary information, Fig. S7a, b). To rigorously trace the fate of human β cells, we used a genetic lineage-tracing approach by transducing human islets with RIP-Cre and CMV-DIO-EGFP lentiviral vectors,^17^ which labeled INS^+^ cells with EGFP. Compared with controls, human islets with excessive zinc treatment exhibited a higher proportion of EGFP^+^GCG^+^ cells, further supporting the hypothesis that excessive zinc induces the conversion of β cells into α cells (Supplementary information, Fig. S7c, d).

To investigate whether zinc accumulation contributes to β-cell identity loss under a chronic diabetic environment, we used *RIP-Cre*; *Rosa26*^*tdTomato*^ HFD-fed mice with zinc supplementation in their drinking water (Fig. 2c). The intraperitoneal glucose tolerance test (i.p. GTT) revealed that HFD-fed mice supplemented with zinc exhibited worse glucose tolerance than HFD-fed mice without extra zinc supplementation (Supplementary information, Fig. S8a). TSQ and ZnT8 staining confirmed that zinc levels were higher in the islets of HFD-fed mice supplemented with zinc than in those of HFD-fed mice without extra zinc supplementation (Fig. 2d, e; Supplementary information, Fig. S8b–d). Consistent with our in vitro results, zinc supplementation significantly reduced the proportion of β cells while increasing the proportions of α cells, bi-hormonal INS^+^GCG^+^ cells, and tdTomato^+^GCG^+^ cells (Fig. 2f–h). These findings demonstrate that excessive zinc supplementation exacerbates β-to-α cell transdifferentiation in HFD-fed mice. In addition, hematoxylin and eosin staining of other major organs (lung, liver, spleen, kidney, and heart), serum biochemistry, and complete blood count revealed no significant differences between HFD-fed mice and HFD-fed mice supplemented with zinc (Supplementary information, Fig. S8e and Table S4).

We next established an acute zinc-overload environment through in situ pancreatic injection of ZnPTO in *RIP-Cre; Rosa26*^*tdTomato*^ mice. After 72 h, immunostaining revealed that the ZnPTO group had significantly lower percentages of β cells and higher percentages of α cells than the controls. In addition, the proportions of bi-hormonal INS^+^GCG^+^ cells and GCG^+^tdTomato^+^ cells were elevated following ZnPTO injection, indicating that β cells underwent transdifferentiation (Supplementary information, Fig. S9a, b).

To minimize the mislabeling of non-β-cell lineages during islet development,^52^ we used an inducible lineage-tracing mouse model (*Ins2-DreER; Rosa26-RSR-ZsGreen*) in which β-cell lineages are labeled with ZsGreen following tamoxifen treatment of adult mice. Using this model, we performed two supplementary experiments: (i) ex vivo islet exposure to excessive zinc (Supplementary information, Fig. S10a–c) and (ii) in situ pancreatic injection of ZnPTO. Both assays provided further evidence that excessive zinc exposure resulted in elevated fractions of GCG^+^ZsGreen^+^ cells, as well as a diminished proportion of β cells accompanied by an increase in α cells (Supplementary information, Fig. S10d–f). In addition, we performed immunostaining for ARX, a key transcription factor in α cells. The results demonstrated that excessive zinc led to an increased proportion of bi-hormonal INS^+^ARX^+^ cells and ARX^+^ZsGreen^+^ cells, further suggesting that zinc accumulation promotes β-to-α cell transdifferentiation (Supplementary information, Fig. S10g–i).

### Disease modeling of β-cell identity loss induced by accumulated zinc in SC-islets

**Fig. 3:**
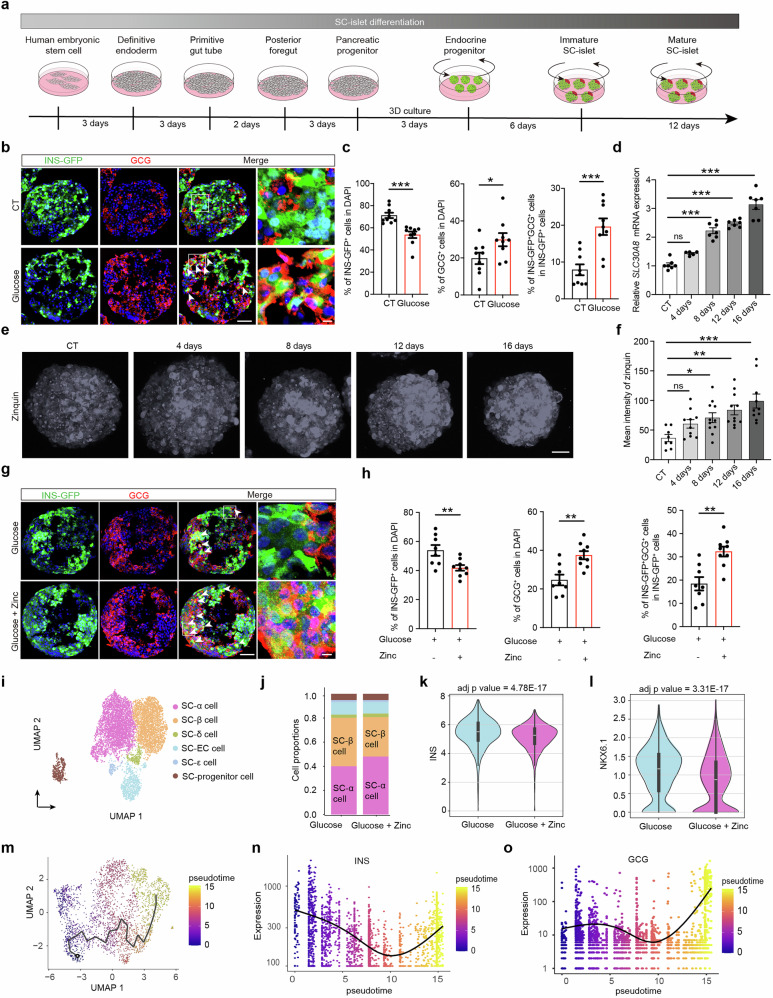
SC-islets, used as a human model, recapitulate β-cell identity loss resulting from zinc accumulation. **a** Schematic flowchart of the SC-islet differentiation process. **b**, **c** Representative immunostaining images (**b**) and corresponding quantification (**c**) showing the percentages of INS-GFP^+^ cells (green) and GCG^+^ cells (red) among the total number of DAPI^+^ cells (blue), as well as the proportion of bi-hormonal INS-GFP^+^GCG^+^ cells among the total INS-GFP^+^ cells, in SC-islets cultured with or without high glucose for 10 days. *n* = 9. Mannitol was used as an osmotic control. White arrows indicate bi-hormonal INS-GFP^+^GCG^+^ cells. Scale bar (low magnification), 50 μm; scale bar (high magnification), 5 μm. **d** qPCR analysis of relative *SLC30A8* (ZnT8) mRNA expression levels at various time points (CT, *n* = 7; 4 days, *n* = 5; 8 days, *n* = 7; 12 days, *n* = 7; and 16 days, *n* = 7) during high-glucose treatment (33 mM). **e**, **f** Representative zinquin staining (**e**) and corresponding measurements of mean fluorescence intensity (**f**) at different time points during high-glucose treatment: CT (*n* = 8), 4 days (*n* = 10), 8 days (*n* = 11), 12 days (*n* = 11), and 16 days (*n* = 10). Scale bar, 50 μm. **g**, **h** Representative immunostaining images (**g**) and corresponding quantification (**h**) showing the percentages of INS-GFP^+^ cells (green) and GCG^+^ cells (red) among the total number of DAPI^+^ cells (blue), as well as the proportion of bi-hormonal INS-GFP^+^GCG^+^ cells among the total INS-GFP^+^ cells in SC-islets cultured in the absence (*n* = 8) or presence (*n* = 9) of excessive zinc under high glucose for 10 days. White arrows indicate bi-hormonal INS-GFP^+^GCG^+^ cells. Scale bar, 50 μm. **i** UMAP analysis showing the distribution of cell types in SC-islets. **j** Proportions of cell types in groups of SC-islets treated with or without excessive zinc under high glucose. **k**, **l** Violin plots showing the distribution of *INS* (**k**) and *NKX6.1* (**l**) expression levels in SC-islets treated with or without excessive zinc under high glucose. **m** Single-cell trajectory of SC-β cells treated with or without excessive zinc under high glucose; cells were ordered by pseudotime along the path from *INS*^high^*GCG*^low^ SC-β cells to *INS*^low^*GCG*^high^ SC-β cells. **n**, **o** Changes in *INS* and *GCG* expression levels along pseudotime. One-way ANOVA with Dunnett’s multiple-comparisons test was used to analyze data in **d**, **f**. Unpaired two-tailed *t*-test was used to analyze data in **c**, **h**. ns not significant; *P < 0.05, **P < 0.01, ***P < 0.001. Data are presented as mean ± SEM. Individual data points are shown for all bar graphs.

Although our findings established a central role for zinc accumulation in β-cell identity loss, the absence of a physiologically relevant human model hampered deeper mechanistic insight into this pathological process. We therefore generated SC-islets for disease modeling to investigate the underlying mechanism using a stepwise SC-islet differentiation method (Fig. 3a).^32^ The MEL1 (*NKX6.1*^*mCherry/mCherry*^*-INS*^*GFP/W*^) hESC reporter line and HUES8 were differentiated into SC-islets. During stepwise differentiation, 96.40% ± 0.83% of the cells expressed both CD117 and CXCR4 at the definitive endoderm stage (Supplementary information, Fig. S11a, c). At the end of the endocrine progenitor stage, 78.77% ± 0.96% of the cells were positive for both NKX6.1 and PDX1 (Supplementary information, Fig. S11b, c). At the mature SC-β cell stage, the SC-islets included appropriate cell types and proportions (70.24% ± 4.56% INS-GFP^+^ cells, 26.38% ± 4.82% GCG^+^ cells, and 7.40% ± 0.89% SST^+^ cells) (Supplementary information, Fig. S11d, e).

To mimic hyperglycemia in patients with T2D, we treated SC-islets with high glucose (33 mM) for 10 days. Immunostaining suggested that high-glucose treatment reduced the percentage of SC-β cells (INS-GFP^+^) while increasing the proportions of SC-α cells (GCG^+^) and bi-hormonal INS-GFP^+^GCG^+^ cells, suggesting a loss of SC-β-cell identity (Fig. 3b, c). Quantitative real-time PCR (qPCR) revealed that expression of *SLC30A8* was upregulated within SC-islets in a time-dependent manner upon glucose treatment (Fig. 3d). Likewise, zinc levels also increased with prolonged high-glucose treatment (Fig. 3e, f), mimicking zinc accumulation in human diabetic islets.

The SC-islets were then exposed to excessive zinc in combination with high glucose to simulate zinc accumulation in diabetic islets, which significantly increased zinc levels in SC-β cells (Supplementary information, Fig. S11f, g). The percentage of INS-GFP^+^ cells was significantly lower in SC-islets treated with excessive zinc than in those without zinc treatment, whereas the percentages of GCG^+^ and bi-hormonal INS-GFP^+^GCG^+^ cells were significantly higher (Fig. 3g, h). To exclude the possibility that increased apoptosis contributed to the reduction in SC-β cells, we performed terminal deoxynucleotidyl transferase biotin-dUTP nick end labeling (TUNEL) staining to evaluate apoptosis in SC-β cells. The results revealed no significant differences in the proportion of apoptotic SC-β cells between the control and high-glucose groups or between the high-glucose-only group and the group treated with both high glucose and excess zinc (Supplementary information, Fig. S12a–d), suggesting that the decrease in SC-β cells is caused primarily by loss of SC-β-cell identity rather than apoptosis. To rigorously trace the fate of SC-β cells, genetic lineage tracing was performed by transducing adherent SC-islets with RIP-Cre and CMV-DIO-EGFP lentiviral vectors, which labeled INS⁺ cells with EGFP. Compared with controls, adherent SC-islets treated with ZnPTO exhibited a higher proportion of EGFP^+^GCG^+^ cells, supporting the hypothesis that excessive zinc induces the conversion of SC-β cells into SC-α cells (Supplementary information, Fig. S12e, f).

By contrast, ZnT8 knockout (KO) SC-islets, which exhibited significantly reduced zinc levels (Supplementary information, Fig. S13a–d), showed a higher proportion of SC-β cells and a lower proportion of SC-α cells upon high-glucose stimulation than wild-type (WT) SC-islets (Supplementary information, Fig. S13e, f). These results demonstrate that zinc accumulation is a significant contributor to SC-β-cell identity loss in a high-glucose environment.

To further investigate the mechanisms underlying β-cell identity loss caused by zinc accumulation, we performed scRNA-seq on SC-islets treated with or without excessive zinc in a high-glucose environment. Cells within the SC-islets were grouped into six distinct clusters (Fig. 3i; Supplementary information, Fig. S14a, b): SC-β cells (*INS, NKX6.1*), SC-α cells (*GCG*, *ARX*), SC-δ cells (*SST*), SC-pancreatic progenitor cells (*SOX9*), SC-ε cells (*GHRL*), and SC-enterochromaffin cells (*FEV*) (Supplementary information, Fig. S14c). Compositional analysis revealed that the proportion of SC-β cells was reduced in SC-islets treated with excessive zinc compared with that in the control group, whereas the proportion of SC-α cells was increased (Fig. 3j). In addition, the mRNA levels of *INS* and *NKX6.1* were reduced in the excessive-zinc group (Fig. 3k, l). These findings indicate that zinc accumulation induces SC-β-cell identity loss in SC-islets. To identify the trajectory of SC-β-cell identity loss, we reordered SC-β cells with Monocle 3 on the basis of pseudotime (Fig. 3m; Supplementary information, Fig. S14d). At the beginning of the trajectory, SC-β cells exhibited high expression of *INS* and low expression of *GCG*. Toward the end of the trajectory, *GCG* expression increased, whereas *INS* expression showed a declining trend (Fig. 3n, o). The expression of *CHGA* remained stable throughout the trajectory, suggesting that endocrine cell identity was preserved (Supplementary information, Fig. S14e). Collectively, disease modeling with SC-islets further confirmed that zinc accumulation induces loss of β-cell identity.

### Zinc accumulation induces ISR to initiate α-cell fate in β cells via the zinc-ATF4-ARX axis

**Fig. 4:**
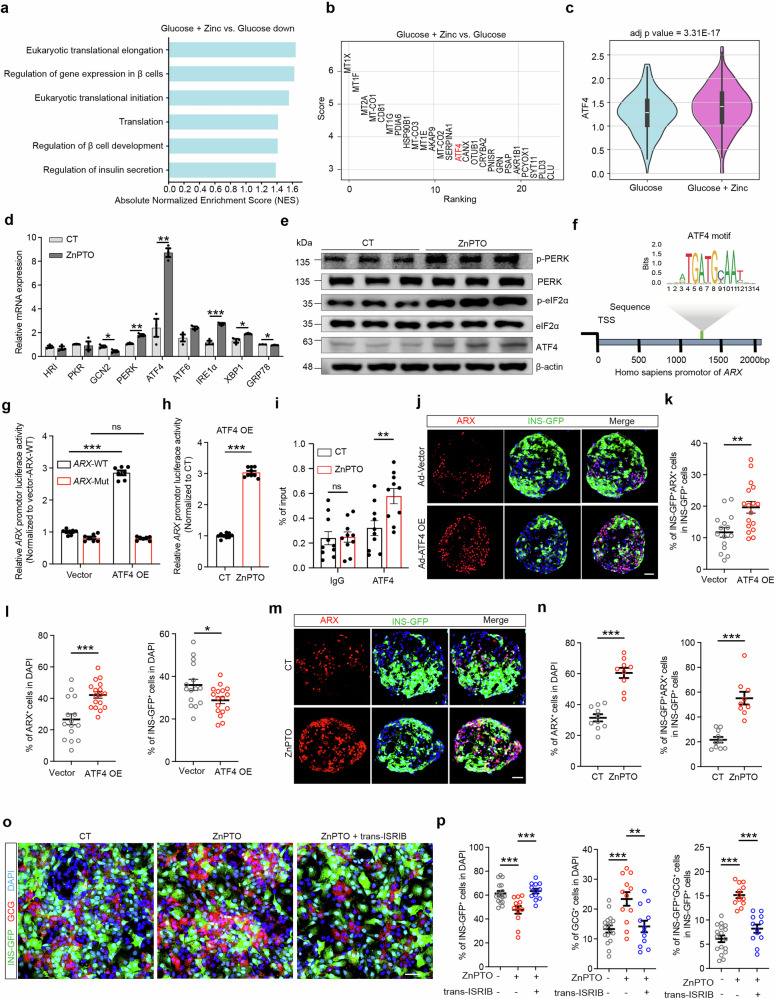
The zinc-ATF4-ARX axis mediates SC-β cell identity loss. **a** Reactome pathway enrichment analysis of DEGs in SC-β cells of SC-islets treated with or without excessive zinc under high glucose using GSEA. **b** Panel showing expression changes of top genes ranked by significance in subcluster 3 of SC-β cells. **c** Violin plots showing *ATF4* expression levels in subcluster 3 of SC-β cells. **d** qPCR analysis showing expression changes of ISR-related genes in SC-islets treated with or without ZnPTO. *n* = 3. **e** Western blot analysis of eIF2α, phosphorylated eIF2α, PERK, phosphorylated PERK, and ATF4 expression in SC-islets treated with or without ZnPTO. *n* = 3. Grayscale analysis of the blots is shown in Supplementary information, Fig. S16a. **f** ATF4 motif analyzed by JASPAR and putative ATF4-binding sites in the proximal promoter region of *ARX*. **g** Dual-luciferase reporter assays measuring relative *ARX* promoter activity using *ARX*-WT and *ARX*-mutation (Mut) constructs in MIN6 cells, with or without *ATF4* OE. *n* = 7. **h** Dual-luciferase reporter assays measuring relative *ARX* promoter activity using *ARX*-WT constructs in MIN6 cells with *ATF4* OE, treated with or without ZnPTO. *n* = 9. **i** Enrichment of ATF4 antibody-bound DNA was measured by qPCR after ChIP assays in SC-islets treated with or without ZnPTO. *n* = 10. **j**–**l** Representative immunofluorescent images (**j**) and quantification (**k**, **l**) of the proportion of INS-GFP^+^ARX^+^ cells among total INS-GFP^+^ cells, as well as the percentages of ARX^+^ cells (red) and INS-GFP^+^ cells (green) among the total number of DAPI^+^ cells (blue) in SC-islets infected with adenovirus carrying either an empty vector (*n* = 15) or an ATF4 OE construct (*n* = 17). Scale bar, 50 μm. **m**, **n** Representative immunofluorescent images (**m**) and quantification (**n**) of the percentages of ARX^+^ cells (red) among the total number of DAPI^+^ cells (blue) and INS-GFP ^+^ARX^+^ cells among total INS-GFP^+^ cells in SC-islets treated with (*n* = 9) or without ZnPTO (*n* = 10). Scale bar, 50 μm. **o**, **p** Representative immunofluorescent images (**o**) and quantification (**p**) of adherent SC-islets under control conditions (*n* = 17), ZnPTO treatment (*n* = 12), and ZnPTO treatment with 7 μM trans-ISRIB (*n* = 12). Scale bar, 25 μm. Two-way ANOVA with Sidak’s multiple-comparisons test was used to analyze data in **g**, **i**. One-way ANOVA with Dunnett’s multiple-comparisons test was used to analyze data in **p**. Unpaired two-tailed *t*-test was used to analyze data in **d**, **h**, **k**, **l**, **n**. ns not significant; *P < 0.05, **P < 0.01, ***P < 0.001. Data are presented as mean ± SEM. Individual data points are shown for all bar graphs.

Gene set enrichment analysis (GSEA) of differentially expressed genes (DEGs) in SC-β cells from the excessive-zinc group versus the control group revealed that pathways associated with β-cell function, including regulation of gene expression in β cells, regulation of β-cell development, and regulation of insulin secretion, were significantly downregulated in the excessive-zinc group. More importantly, translation-related pathways were also suppressed, including eukaryotic translational elongation, eukaryotic translational initiation, and translation (Fig. 4a). Upon further subclustering of SC-β cells, we identified a subcluster (subcluster 3) characterized by high *GCG* expression (Supplementary information, Fig. S15a–d). In this subcluster, we observed activation of the transcription factor ARX. SCENIC-based analysis of transcription factor activity confirmed that this shift in gene expression was specifically associated with activation of ARX in these cells (Supplementary information, Fig. S15e).^53,54^ Notably, within the β cells of the excessive-zinc group, we observed upregulation of *ARX* transcriptional activity, accompanied by concurrent increases in *GCG* and *ARX* expression and decreases in *INS* and *NKX6.1* expression (Supplementary information, Fig. S15f, g).

Analysis of DEGs in SC-β cells revealed that the ISR-related downstream transcription factor *ATF4* ranked among the top 20 DEGs in subcluster 3 following treatment with both glucose and excessive zinc compared with glucose treatment alone (Fig. 4b, c). Upregulation of *ATF4* and downregulation of translational initiation are hallmarks of the ISR,^55^ we therefore analyzed the expression of ISR-related genes. Dot-plot analysis revealed increased expression of ISR-associated genes, including the eIF2α kinase gene *EIF2AK3* (also known as *PERK*), in SC-β cells treated with excessive zinc compared with controls (Supplementary information, Fig. S15h), suggesting that excessive zinc activates the ISR pathway.

To confirm this possibility, we treated SC-islets with ZnPTO to examine the expression of ISR-related kinases and their downstream transcription factors. Both qPCR and western blot analyses revealed that PERK and ATF4 were significantly upregulated in SC-islets treated with ZnPTO (Fig. 4d, e; Supplementary information, Fig. S16a). Western blot analysis also revealed that excessive zinc induced phosphorylation of eIF2α in these SC-islets (Fig. 4e; Supplementary information, Fig. S16a), suggesting activation of the ISR. By contrast, both the zinc chelator TPEN (N,N,N′,N′-tetrakis (2-pyridylmethyl) ethylenediamine)^56^ and genetic depletion of ZnT8 (ZnT8 KO) reduced eIF2α phosphorylation in SC-islets (Supplementary information, Fig. S16b, c). To determine whether zinc, rather than other ions such as copper or iron, specifically activates the ISR by inducing eIF2α phosphorylation, we assessed eIF2α phosphorylation using metal ionophores. The results showed that CuCl_2_:elesclomol (a copper ionophore) and erastin (a reagent that increases intracellular iron concentration) did not affect eIF2α phosphorylation, suggesting that zinc specifically induces eIF2α phosphorylation in β cells compared with copper and iron (Supplementary information, Fig. S16d).

To investigate whether upregulation of ATF4 regulates the expression of genes involved in α-cell fate, we consulted the JASPAR database (https://jaspar.genereg.net/matrix/MA0833.2/),^57,58^ which revealed a potential transcription factor-binding site for ATF4 in the upstream region of ARX, an α cell-specific transcription factor (Fig. 4f). On the basis of this prediction, we performed dual-luciferase reporter assays, which demonstrated that ATF4 bound directly to the *ARX* promoter region and significantly enhanced its transcriptional activity (Fig. 4g). We also included a ZnPTO treatment group in the dual-luciferase reporter assay. The results demonstrated that ARX-luciferase activity was significantly higher in the ZnPTO-treated group than in the control group, confirming that increased zinc levels enhance ATF4-driven activation of the *ARX* promoter (Fig. 4h). Chromatin immunoprecipitation–quantitative PCR (ChIP–qPCR) further demonstrated enhanced ATF4 binding at the ARX promoter region following ZnPTO treatment (Fig. 4i).

To determine whether ATF4 upregulation activates *ARX* expression at the protein level, ATF4 was overexpressed in SC-islets. The results revealed that the percentage of SC-β cells decreased, whereas the percentages of ARX^+^ cells and INS-GFP^+^ARX^+^ cells increased significantly, suggesting misexpression of ARX in SC-β cells (Fig. 4j–l). Consistent with these results, ATF4 overexpression (OE) ultimately resulted in an increased percentage of SC-α cells in SC-islets (Supplementary information, Fig. S16e, f). We observed a similar trend in human primary islets with ATF4 OE, which showed a reduction in β cells and an increase in α cells (Supplementary information, Fig. S16g, h). Moreover, ZnPTO treatment caused ARX upregulation and misexpression in SC-β cells and human primary β cells (Fig. 4m, n; Supplementary information, Fig. S16i, j).

We next treated SC-islets with the ISR inhibitor trans-ISRIB^59^ to determine whether inhibition of zinc-induced ATF4 expression could prevent β-cell identity loss. As expected, the transdifferentiation process was inhibited in SC-islets treated with both trans-ISRIB and ZnPTO, as evidenced by a reduced proportion of SC-α cells compared with that in SC-islets treated with ZnPTO alone (Fig. 4o, p).

Collectively, these results demonstrate that accumulated zinc triggers the ISR with elevated ATF4 expression in SC-β cells. This, in turn, initiates expression of the α-cell-specific transcription factor ARX, resulting in conversion of β cells to α cells and formation of a zinc-ATF4-ARX regulatory axis.

### Primary islets and SC-islets undergo β-cell identity loss accompanied by zinc accumulation after transplantation

**Fig. 5:**
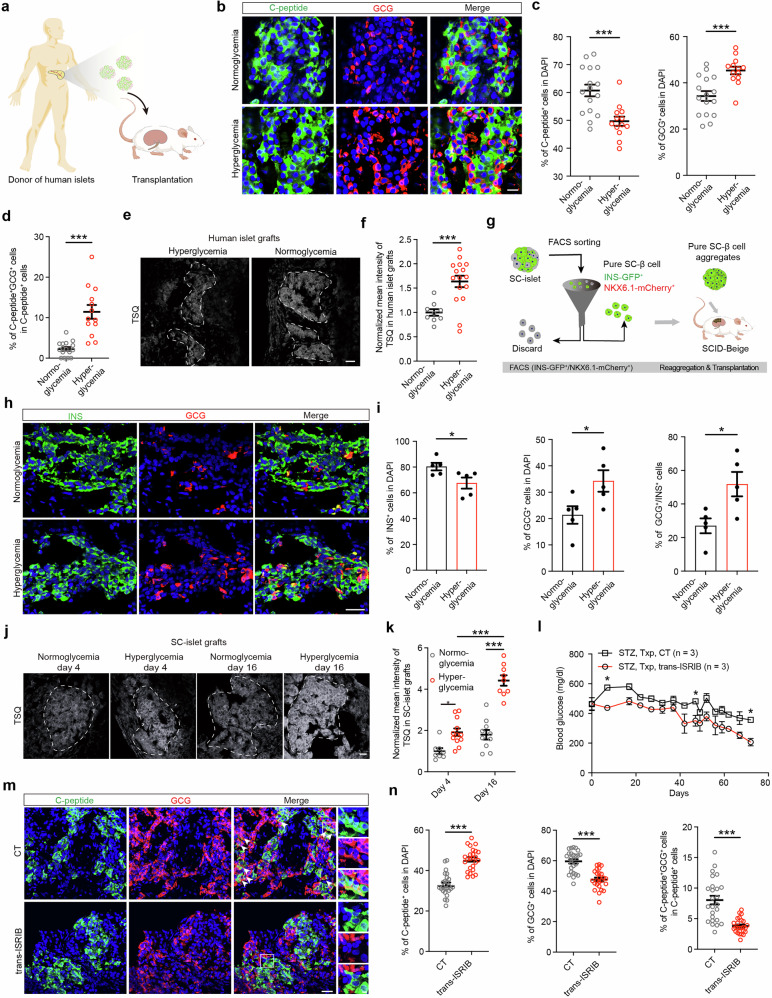
β cells undergo identity loss after transplantation into diabetic animals. **a** Schematic diagram illustrating the transplantation of human primary islets into mice. The diagram was created with figdraw.com. **b**–**d** Representative immunofluorescent images (**b**) and quantification (**c**, **d**) of the percentages of C-peptide^+^ cells (green) and GCG^+^ cells (red) among the total number of DAPI^+^ cells, as well as the proportion of bi-hormonal C-peptide^+^GCG^+^ cells among the total C-peptide^+^ cells, in human islet grafts from normoglycemic mice (*n* = 16) and hyperglycemic mice (*n* = 13). Scale bar, 10 μm. **e**, **f** Representative TSQ staining images (**e**) and normalized mean intensity measurements (**f**) of human islet grafts transplanted into normoglycemic (*n* = 9) and hyperglycemic (*n* = 16) mice. Scale bar, 25 μm. **g** Schematic diagram of the method used to obtain pure SC-β cell aggregates from SC-islets by FACS. The diagram was created with figdraw.com. **h**, **i** Representative immunofluorescent images (**h**) and quantification (**i**) of the percentages of INS^+^ cells (green) and GCG^+^ cells (red) among the total number of DAPI^+^ cells (blue), as well as the ratio of GCG^+^ cells to INS^+^ cells, in pure β-cell grafts from normoglycemic mice and hyperglycemic mice. *n* = 5. Scale bar, 25 μm. **j**, **k** Representative TSQ staining images (**j**) and normalized mean intensity measurements (**k**) of grafts at day 4 and day 16 following SC-islet transplantation into normoglycemic mice (day 4, *n* = 8; day 16, *n* = 10) and hyperglycemic mice (day 4, *n* = 12; day 16, *n* = 9). Scale bar, 25 μm. **l** Blood glucose measurements in randomly fed STZ-induced hyperglycemic mice transplanted with SC-islets with or without trans-ISRIB injection (Txp, transplantation; black line: STZ, SC-islet Txp, *n* = 3; red line: STZ, SC-islet Txp with trans-ISRIB injection, *n* = 3). **m**, **n** Representative immunofluorescent images (**m**) and quantification (**n**) of the percentages of C-peptide^+^ cells (green) and GCG^+^ cells (red) among the total number of DAPI^+^ cells (blue), as well as the proportion of bi-hormonal C-peptide^+^GCG^+^ cells among the total C-peptide^+^ cells, in SC-islet grafts from hyperglycemic mice with or without trans-ISRIB injection. *n* = 25. White arrows indicate bi-hormonal C-peptide^+^GCG^+^ cells. Scale bar (low magnification), 25 μm; scale bar (high magnification), 5 μm. Two-way ANOVA with Sidak’s multiple-comparisons test was used to analyze data in **k**, **l**. Unpaired two-tailed *t* test was used to analyze data in **c**, **d**, **f**, **i**, **n**. ns not significant; *P < 0.05, ***P < 0.001. Data are presented as mean ± SEM. Individual data points are shown for all bar graphs.

β-cell loss frequently occurs after transplantation, making it crucial to investigate whether loss of identity contributes to this reduction.^60^ To address this possibility, human primary islets were transplanted into normoglycemic mice or STZ-induced hyperglycemic mice for 5 days (Fig. 5a). Immunofluorescence staining revealed a significant reduction in the proportion of β cells and significant increases in the proportions of α cells and bi-hormonal INS^+^GCG^+^ cells in human islet grafts from hyperglycemic mice compared with those from normoglycemic mice (Fig. 5b–d), suggesting that β cells underwent identity loss after transplantation. Moreover, TUNEL staining revealed no significant differences between normoglycemic mice and hyperglycemic mice in the percentage of apoptotic β cells in grafts, excluding the possibility that increased apoptosis was responsible for the reduction in β cells (Supplementary information, Fig. S17a, b). Similar to β cells in primary islets from patients with T2D, β cells in human islet grafts from hyperglycemic mice exhibited significantly higher ZnT8 expression than those from normoglycemic mice (Supplementary information, Fig. S17c, d), consistent with the zinc accumulation patterns observed using TSQ staining (Fig. 5e, f).

To further investigate whether SC-β-cell identity loss occurs after transplantation, we used a strategy in which pure SC-β cells were sorted and collected on the basis of their double-positive fluorescence for INS-GFP and NKX6.1-mCherry (Fig. 5g). The sorted SC-β cells were then reaggregated into pure SC-β-cell clusters. Flow cytometry analysis and immunofluorescence staining showed that > 98.8% of the sorted SC-β cells were double positive for INS-GFP and NKX6.1-mCherry, whereas SC-α cells accounted for less than 1% of the population (Supplementary information, Fig. S18a–c). Next, pure SC-β-cell aggregates were transplanted into normoglycemic mice or STZ-induced hyperglycemic mice for 5 days. Under hyperglycemic conditions, we observed a significant decrease in the percentage of SC-β cells and an increase in the percentage of SC-α cells in pure SC-β cell aggregates (Fig. 5h, i). These findings suggest that GCG-expressing cells originated from SC-β cells. In addition, TSQ and ZnT8 staining revealed increased zinc levels in grafts of SC-islets from hyperglycemic mice compared with those from normoglycemic mice (Fig. 5j, k; Supplementary information, Fig. S18d, e), indicating that β-cell identity loss is associated with zinc accumulation after transplantation.

To determine whether prevention of zinc-ATF4-ARX-induced SC-β-cell identity loss could improve glycemic control in SC-islets, we transplanted SC-islets into hyperglycemic mice and administered the ISR inhibitor trans-ISRIB via i.p. injection once a day. Mice transplanted with SC-islets and administered trans-ISRIB exhibited improved blood glucose restoration (Fig. 5l). The i.p. GTT and in vivo glucose-stimulated insulin secretion (GSIS) assays showed that mice treated with trans-ISRIB had improved glucose tolerance and enhanced human insulin secretion (Supplementary information, Fig. S18f, g). Immunofluorescence staining showed a significant increase in SC-β cells, along with a decrease in SC-α cells and bi-hormonal INS^+^GCG^+^ cells, in grafts from mice treated with trans-ISRIB (Fig. 5m, n). These data reveal that both primary islets and SC-islets undergo identity loss accompanied by zinc accumulation after transplantation and that protecting SC-β cells from identity loss by inhibiting the ISR can effectively improve glycemic control in vivo.

### Genetic depletion of ZnT8 protects SC-β cells from identity loss

Our study revealed that zinc levels were significantly reduced in ZnT8 LOF SC-β cells. To investigate whether modulation of zinc levels by ZnT8 LOF prevents SC-β-cell identity loss after transplantation, WT and ZnT8 KO SC-islets were implanted under the kidney capsules of hyperglycemic mice. Five days after transplantation, we performed immunofluorescence staining to examine the cell composition of SC-islets in the grafts. Compared with ZnT8 KO SC-islets, WT SC-islets in the grafts exhibited a significant decrease in INS^+^ cells and an increase in GCG^+^ cells (Supplementary information, Fig. S19a, b). This change occurred even though the SC-β-cell compositions were nearly identical in both types of SC-islets before transplantation (Supplementary information, Fig. S19c, d), indicating that zinc reduction mediated by ZnT8 LOF helps preserve SC-β-cell identity after transplantation.

We used an mCherry-based tracing strategy to track SC-β-cell fate, in which *mCherry* was linked to *NKX6.1* via a 2A linker and *GFP* was expressed under the *INS* promoter (*NKX6.1*^*mCherry/mCherry*^*-INS*^*GFP/W*^). Unlike other pancreatic endocrine cells, β cells are defined by *NKX6.1* expression, which distinguishes them from bi-hormonal and α cells. Bi-hormonal cells express *INS* and *GCG* but lack *NKX6.1*, α cells express *GCG* without *NKX6.1*, and pure β cells co-express *NKX6.1* and *INS*.^61,62^ Furthermore, mCherry can be inherited by SC-α cells transdifferentiated from SC-β cells, as it is a stable fluorescent protein that persists long-term, especially in SC-β cells (Supplementary information, Fig. S19e).^63,64^ Immunostaining for mCherry was performed alongside staining for GCG and NKX6.1 in SC-islets. SC-β cells were labeled INS-GFP^+^, NKX6.1^+^, and mCherry^+^ but GCG^–^. By contrast, original SC-α cells were labeled GCG^+^, INS-GFP^–^, NKX6.1^–^, and mCherry^–^, whereas SC-α cells transdifferentiated from SC-β cells were labeled GCG^+^, INS-GFP⁻, NKX6.1^–^, and mCherry^+^, indicating inheritance of mCherry from SC-β cells despite loss of the β-cell identity marker *NKX6.1* (Supplementary information, Fig. S19f). Using this strategy, we found that SC-β cells (INS^+^mCherry^+^GCG^–^) transdifferentiated into SC-α cells (INS^−^mCherry^+^GCG^+^) in WT SC-islets. By contrast, ZnT8 LOF inhibited this process in transplanted SC-islets under hyperglycemic conditions (Supplementary information, Fig. S19g, h).

### Anisomycin protects SC-β cells from identity loss

**Fig. 6:**
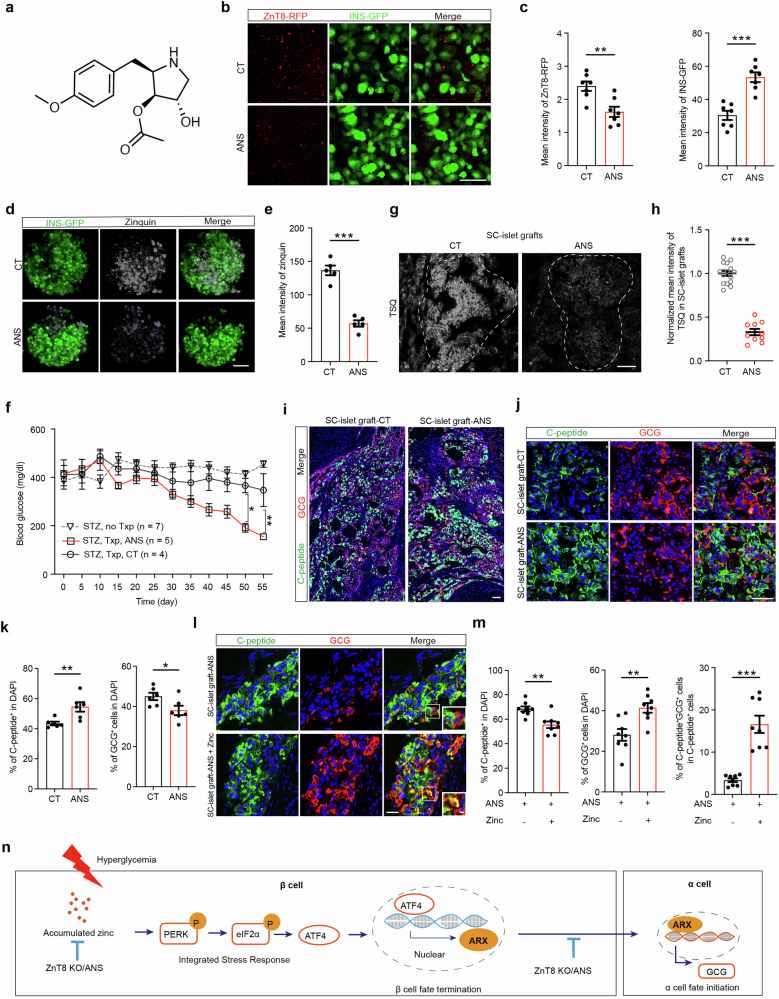
Anisomycin safeguards β cells against identity loss by inhibiting zinc accumulation. **a** Chemical structure of anisomycin ([(2R,3S,4S)-4-hydroxy-2-[(4-methoxyphenyl) methyl]pyrrolidin-3-yl] acetate). **b**, **c** Representative images (**b**) and measurements (**c**) of mean intensity of ZnT8-RFP and INS-GFP in adherent SC-islets treated with or without ANS. *n* = 7. Scale bar, 50 μm. **d**, **e** Representative images (**d**) and measurements (**e**) of zinquin intensity in SC-islets treated with or without ANS. *n* = 5. Scale bar, 50 μm. **f** Blood glucose levels in randomly fed STZ-induced hyperglycemic mice transplanted with SC-islets, with or without ANS treatment (red line: STZ, MEL1-WT SC-islet Txp, with ANS injection, *n* = 5; gray line: STZ, MEL1-WT SC-islet Txp with control, *n* = 4; gray dotted line: STZ, no Txp, *n* = 7). **g**, **h** Representative images (**g**) and quantification of normalized mean TSQ fluorescence intensity (**h**) in SC-islet grafts transplanted into hyperglycemic mice with control (*n* = 16) or ANS (*n* = 10) treatment. Scale bar, 50 μm. **i**, **j** Representative immunofluorescent images of C-peptide (green) and GCG (red) in SC-islet grafts from hyperglycemic mice with or without ANS administration, shown at low (**i**) and high (**j**) magnification. Scale bars, 50 μm. **k** Quantification of the percentages of C-peptide^+^ cells and GCG^+^ cells among the total number of DAPI^+^ cells in SC-islet grafts corresponding to (**j**). *n* = 6. **l**, **m** Representative immunofluorescence images (**l**) and quantification (**m**) of the percentages of C-peptide^+^ cells and GCG^+^ cells among the total number of DAPI^+^ cells, as well as the proportion of bi-hormonal C-peptide^+^GCG^+^ cells among the total C-peptide^+^ cells, in SC-islet grafts from hyperglycemic mice treated with ANS alone or in combination with zinc supplementation. *n* = 8. Scale bar (low magnification), 25 μm; scale bar (high magnification), 5 μm. **n** Schematic diagram of the mechanism by which accumulated zinc induces ISR, thereby triggering identity loss of pancreatic β cells. Two-way ANOVA with Sidak’s multiple-comparisons test was used to analyze data in **f**. Unpaired two-tailed *t*-test was used to analyze data in **c**, **e**, **h**, **k**, **m**. ns not significant; *P < 0.05, **P < 0.01, ***P < 0.001. Data are presented as mean ± SEM. Individual data points are shown for all bar graphs.

Our results demonstrate that excessive zinc induces conversion of β cells to α cells and that modulation of zinc levels by ZnT8 LOF prevents the loss of β-cell identity. This finding raises possibility that pharmacological modulation of zinc accumulation could also contribute to preservation of β-cell identity. Through a compound screen targeting ZnT8-mediated zinc transport, we identified anisomycin (ANS), a multifunctional drug (Fig. 6a), which at a 25 nM dose significantly reduced ZnT8 translation in SC-islets engineered with an RFP tag fused to the ZnT8 C terminus (Fig. 6b, c; Supplementary information, Fig. S20a). Notably, ANS is generally applied as a protein synthesis inhibitor at 10 μM,^65^ which is 400-fold greater than the dose used in our study. To determine whether ANS has an inhibitory effect on protein synthesis in SC-islets at a concentration of 25 nM, we performed an O-propargyl-puromycin (OPP)-labeled nascent polypeptide synthesis assay.^66,67^ The mean intensity of OPP fluorescence revealed that total protein synthesis was not impaired in the 25 nM ANS treatment group (Supplementary information, Fig. S20b, c). In addition, ANS treatment significantly increased INS-GFP expression in SC-islets (Fig. 6b, c). These results suggest that 25 nM ANS does not inhibit general protein synthesis, consistent with previous reports.^65^ Moreover, 25 nM ANS treatment did not increase cell death in SC-islets (Supplementary information, Fig. S20d, e).

Since ANS significantly suppressed ZnT8 expression, we next examined whether ANS could inhibit zinc accumulation. The results revealed that ANS treatment significantly reduced zinc levels in SC-islets compared with the control group (Fig. 6d, e). To further assess whether ANS preserves β-cell identity, the compound was added to the culture medium of SC-islets under high-glucose conditions. As a result, the percentage of SC-β cells (INS-GFP^+^) increased in the ANS-treated group compared with the high-glucose group without ANS treatment, whereas the proportion of SC-α cells (GCG^+^) was significantly reduced (Supplementary information, Fig. S20f, g). These results show that, similar to ZnT8 LOF, ANS effectively prevents conversion of SC-β cells to SC-α cells in SC-islets by chemically targeting zinc accumulation.

We next investigated whether SC-islets treated with ANS could improve glycemic restoration in hyperglycemic mice by preventing SC-β-cell transdifferentiation under hyperglycemic conditions in vivo. To test the efficacy of ANS in vivo, SC-islets were treated with 25 nM ANS for 72 h and transplanted into hyperglycemic mice. ANS was then administered at 40 mg/kg every 5 days to hyperglycemic mice transplanted with SC-islets pretreated with ANS. In parallel, vehicle was administered to hyperglycemic mice transplanted with SC-islets as a control. Hyperglycemic mice transplanted with SC-islets and treated with ANS showed better glycemic control than those without ANS treatment (Fig. 6f). To determine whether ANS treatment protects SC-β-cell identity in hyperglycemic mice, the kidney grafts were removed and examined. The results revealed that ANS administration enhanced C-peptide expression while effectively reducing ZnT8 expression and zinc accumulation in SC-islet grafts (Fig. 6g, h; Supplementary information, Fig. S20h, i). Moreover, immunostaining showed an increase in SC-β cells and a decrease in SC-α cells in ANS-treated SC-islet grafts (Fig. 6i–k). We also treated hyperglycemic mice bearing SC-islet grafts with ANS while simultaneously providing zinc-supplemented drinking water. TSQ staining revealed that zinc supplementation reversed the ANS-induced reduction in zinc accumulation within the grafts (Supplementary information, Fig. S20j, k). Furthermore, in grafts from mice given zinc-supplemented water, the proportion of SC-β cells decreased, whereas the proportions of SC-α cells and bi-hormonal INS^+^GCG^+^ cells increased (Fig. 6l, m), suggesting that zinc supplementation antagonizes the protective effect of ANS on SC-β-cell identity. Collectively, these results suggest that ANS effectively preserves the identity of SC-β cells by alleviating zinc accumulation in hyperglycemic mice. Notably, histological analysis of major organs and serum biochemistry revealed no apparent toxicity in ANS-treated mice compared with controls (Supplementary information, Fig. S20l and Table S5).

The above results reveal that excessive zinc activates the ISR with elevated ATF4 expression in SC-β cells. This initiates expression of the α cell-specific transcription factor ARX and thus leads to conversion of SC-β cells to SC-α cells, forming a zinc-ATF4-ARX regulatory axis. Moreover, ZnT8 depletion and ANS effectively safeguard SC-β cells from identity loss and improve glycemic control by inhibiting zinc accumulation (Fig. 6n).

## Discussion

Recent studies have suggested that identity loss is a critical cause of β-cell failure in T2D.^6–10^ However, the mechanism of β-cell identity loss in patients with T2D remains unknown. In this study, we identified zinc accumulation as a key factor driving β-cell identity loss under diabetic conditions. Using SC-islets for disease modeling, we found that excessive zinc triggered the ISR, leading to β-cell identity loss through a zinc-ATF4-ARX signaling axis. Moreover, we demonstrated that both primary human islets and SC-islets transplanted into hyperglycemic animals experienced β-cell identity loss together with zinc accumulation. These findings highlight the detrimental effect of zinc accumulation on β-cell identity.

Recent breakthroughs have shed light on the pivotal role of metals in cell death. Iron-induced ferroptosis, for example, is characterized by the accumulation of reactive oxygen species and lipid peroxides, leading to failure of glutathione-dependent antioxidants and consequent cell death.^68^ In addition, the discovery of copper-induced cell death has broadened our understanding of metal-mediated cell death. Tsvetkov et al. revealed that copper specifically binds to lipoylated proteins of the tricarboxylic acid cycle, leading to cellular proteotoxicity and consequent cell death.^69^ However, the spectrum of metal-related cell failure extends beyond mere cell death to encompass loss of cell identity. The effects of metals on cell identity remain largely unknown. In this study, we discovered that zinc accumulation leads to identity loss of pancreatic β cells. Whether zinc-induced cellular identity loss contributes to other diseases caused by functional cell failure besides diabetes warrants further investigation.

Deciphering the molecular mechanisms that underlie β-cell identity loss has been a major challenge in diabetes research. β cells are highly susceptible to the ISR mediated by eIF2α phosphorylation, which causes β-cell failure.^55^ Recent structural analyses revealed that the eIF2 complex possesses a zinc-binding site on eIF2β,^70^ which forms part of the nucleotide-binding pocket.^71^ On the basis of structural analysis, it is possible that zinc binding to the eIF2 complex promotes the stability of the inactive eIF2B–eIF2-P (phosphorylated) complex by stabilizing the structure of eIF2β and insulating phosphorylated eIF2α from phosphatase hydrolysis. Dephosphorylation of eIF2-P is a tightly regulated process involving two phosphatase complexes in mammalian cells. Both phosphatase complexes contain a common catalytic core, protein phosphatase 1 (PP1).^72^ Another study showed that zinc can inhibit phosphatases and lead to persistent kinase activation.^73^ Thus, excessive zinc may also inhibit PP1-mediated dephosphorylation, resulting in accumulation of eIF2α phosphorylation. Here, we demonstrated that excessive zinc leads to identity loss of pancreatic β cells through induction of eIF2α phosphorylation and activation of the ISR. Further investigations are warranted to characterize the precise role of zinc in modulating eIF2α phosphorylation and ISR dynamics.

On the basis of this mechanistic understanding, we investigated the potential for ANS to serve as a protective agent for β-cell identity by inhibiting zinc transport. ANS is a bacterial pyrrolidine antibiotic isolated from *Streptomyces roseochromogenes* and *Streptomyces griseolus*.^74^ As a multifunctional drug, its effects vary at different doses. ANS is known mainly as an inhibitor of protein synthesis in eukaryotic organisms.^75^ It suppresses eukaryotic peptidyl transferase activity with a K_i_ (inhibition constant) of 6.5 ×  10^−7^ M.^76^ Approximately 10 μM ANS inhibits 95% of protein synthesis in HeLa cells.^75^ In addition, ANS (0.1 μM) activates mitogen-activated protein kinase cascades in mammalian cells.^77^ Moreover, ANS also induces protein degradation.^65^ At 37 μM, ANS accelerates degradation of phosphorylated connexin 43 (Cx43) by activation of p38.^78^ Exposure to 0.5–1 μM ANS can lead to aryl hydrocarbon receptor (AhR) proteasome-mediated degradation in the human breast epithelial cell line MCF10A.^79^ Interestingly, ANS (< 100 nM) promotes survival of PC12 cells and exhibits immunosuppressive or antitumor effects.^80–82^ The in vivo LD50 (lethal dose 50%) of intravenously administered ANS in mice is 451.1 μmol/kg.^83^ A high dose (226.2 μmol/kg) of ANS significantly decreases body weight, and marked inflammatory reactions are observed in the lungs, liver, and kidneys. However, relatively low doses of ANS (56.5 μmol/kg) are not associated with significant adverse effects at therapeutically effective levels.^83^ In this study, we revealed a new function of ANS in protecting SC-β-cell identity by modulating zinc levels, suggesting a potential approach to improving the efficacy of cell-replacement therapy for diabetes.

## Materials and methods

### Maintenance of hESCs

Before hESCs were seeded, dishes were coated with Matrigel diluted to 1:80 (Corning, #354277) and seeded with irradiated mouse embryonic fibroblasts (MEFs). hESCs were cultured in hESC maintenance medium containing DMEM/F12 (Gibco, #11330-082), 20% knockout serum replacement (KOSR) (Gibco, #10828028), 1 mM non-essential amino acids (Gibco, #11140-050), 1% L-glutamine (Gibco, #25-005-CI), 0.1 mM β-mercaptoethanol (Gibco, #21985023), and 10 ng/mL bFGF (R&D System, #2v33-FB/CF). hESCs were maintained at 37 °C with 5% CO_2_ and 5% O_2_; they were dissociated into single-cell suspensions by incubation with TrypLE Express Enzyme (Gibco, #12604-021) and passaged every 3–4 days. Checks for mycoplasma contamination were performed regularly to ensure the integrity of the cells. The ZnT8 KO hESC lines (MEL1 and HUES8) were generated by Dr. Qing Ma, and the MEL1 ZnT8 reporter line was established by co-author Zhaoyue Wang.

### In vitro differentiation of SC-islets derived from hESCs

Mature SC-islets were obtained using a protocol adapted from previous studies.^32^ The differentiation process included seven stages and lasted for 35 days. Stages 1–4 involved planar culture to generate pancreatic progenitors, and stages 5–7 involved 3D culture to generate mature SC-islets (see details in Supplementary information, Table S6).

### ANS-related assay

#### Cell assay

ANS (TargetMol, #T6758) was dissolved in DMSO (Sigma‒Aldrich, #D2650) to prepare a 10 mM stock solution (kept in the dark). The stock solution was aliquoted and stored at –80 °C. Repeated freeze–thaw cycles were avoided. The 10 mM ANS stock solution was diluted with DMSO to a 50 μM working solution. This working solution was added to the medium at a ratio of 1:2000 to achieve a final ANS concentration of 25 nM. The SC-islets were cultured with 25 nM ANS at 37 °C in an incubator with 5% CO_2_ and 5% O_2_ for the experiments.

#### Mouse treatment

ANS was dissolved in a solution consisting of 95% DPBS and 5% DMSO. Mice received i.p. injections of ANS (40 mg/kg) every 5 days for 8 weeks post transplantation. Animals were randomly divided into vehicle and ANS groups.

### Immunohistochemistry

#### Cells

Cell aggregates were rinsed with PBS and fixed with 4% paraformaldehyde (PFA, Sangon Biotech, #E672002-0500) overnight at 4 °C. After removal of PFA, the cell aggregates were rinsed with 0.5% BSA solution (PBS containing 0.5% BSA) (Proliant, #69100), embedded in optimal cutting temperature (OCT) compound, and stored at –80 °C to obtain frozen sections. The cell aggregates were sliced into 8-μm sections on slides using a cryomicrotome. The slides were incubated with a blocking solution (PBST containing 5% donkey serum) for 1 h at room temperature (RT) and incubated with primary antibodies at appropriate dilutions overnight at 4 °C. Secondary antibodies were added for 45 min at RT after 3 rinses with PBST. Cell nuclei were stained with DAPI dihydrochloride, and images were captured with an Olympus FV3000 microscope.

#### Human pancreas tissue

Paraffin sections of human pancreatic tissue (approval number: CHEC2024-109) were heated at 60 °C for 60 min. For dewaxing and rehydration, the sections were treated with xylene (10 min) 3 times, absolute ethanol (5 min) twice, 95% ethanol for 5 min, 75% ethanol for 5 min, and distilled water (5 min) twice. The sections were then immersed in antigen retrieval solution, preheated by microwaving, and cooled to RT. The remaining steps were performed according to the manufacturer’s instructions for the BAS kit (Alpha X (Beijing) Biotech, BXT34100011). BMI and blood glucose data for the donors are provided in Supplementary information, Tables S2, S3.

#### Mouse tissue

Tissues were washed with PBS, trimmed of excess parts, and fixed with 4% PFA for 1 h at 4 °C. The fixed tissues were washed once with PBS and then dehydrated overnight in a 30% sucrose solution (PBS containing 30% sucrose). After dehydration, the tissues were embedded in OCT and stored at –80 °C to obtain frozen sections. The remaining steps were performed according to the manufacturer’s instructions for the BAS kit (Alpha X (Beijing) Biotech, BXT34100011).

### TUNEL staining

Slides were permeabilized with proteinase K (20 μg/mL, 10 min), equilibrated (1× buffer, 25 min), and labeled with a TdT enzyme/YSFluor488-dUTP mix (YEASEN Biotechnology, 40306ES20) (37 °C, 60 min). After PBS washes, the slides were incubated with primary antibodies at appropriate dilutions overnight at 4 °C. After three rinses with PBST, secondary antibodies were added for 45 min at RT. Cell nuclei were stained with DAPI dihydrochloride, and images were obtained using an Olympus FV3000 microscope.

### Luciferase assay

MIN6 cells (Zhong Qiao Xin Zhou Biotechnology, #ZQ0462) were seeded in 24-well plates and cultured in high-glucose DMEM (BasalMedia Technologies, #L110KJ) supplemented with 15% fetal bovine serum. To generate the ARX-Mut construct, a 14-bp sequence at the ATF4 binding site within the ARX promoter was deleted, introducing a frameshift mutation. The mutated promoter sequence was cloned upstream of the luciferase reporter (pGL4.10-ARX-Mut-luciferase), alongside the ARX-WT construct (pGL4.10-ARX-WT-luciferase), to serve as a control for ATF4-mediated ARX promoter activation. Each well was co-transfected with 200 ng of the pGL4.10-ARX promoter construct (WT or 14-bp deletion mutant) and 200 ng of either pcDNA3.1-ATF4-3×FLAG or the empty vector using Lipofectamine 2000. To normalize transfection efficiency, 20 ng of a *Renilla* luciferase plasmid was co-transfected. At 48 h post transfection, firefly and *Renilla* luciferase activities were measured using a dual-luciferase reporter assay system (YEASEN Biotechnology, #11402ES60).

### ZnPTO treatment

ZnPTO powder (Sigma–Aldrich, #H6377-10G) was dissolved in DMSO to create a 20 mM stock solution, which was stored at –80 °C. For ZnPTO treatment of SC-islets, SC-islets were cultured in 2 mL of S7 medium supplemented with 3 μM ZnPTO in an incubator (37 °C, 5% CO_2_) for 24 h. Samples in the control group were treated with an equivalent volume of DMSO. For adherent SC-islets, SC-islets were first dissociated with 0.25% trypsin and seeded onto 33-mm confocal dishes (Beyotime, #FCFC020-150pcs) coated with Matrigel diluted to 1:3. ZnPTO was then added to a final concentration of 3 µM, and cells were cultured in an incubator (37 °C, 5% CO_2_) for 24 h. For in situ pancreatic injection in mice, the pancreas and spleen of anesthetized mice were exposed, and a single dose of ZnPTO (5 mM) was microinjected (200 μL) into the tail of the pancreas. After 72 h, the pancreas was extracted, and immunostaining was performed.

### Adenovirus generation

A pENTR-gene-mCherry entry clone was generated for the gene of interest, and the pAd/CMV/V5-DEST Gateway kit (Invitrogen, #V49320) was then used to perform the LR recombination reaction to create a pAd/CMV/V5-gene-mCherry adenoviral expression clone. The pAd/CMV/V5-gene-mCherry adenoviral expression clone was digested with PacI (New England Biolabs, #R0547S) to expose the inverted terminal repeat sequences (ITRs), and the digested clone was purified by phenol/chloroform extraction followed by ethanol precipitation. 293A cells were then transfected with the adenoviral expression clones using Lipofectamine 2000 (Thermo Fisher Scientific, #11668019), and crude adenoviral lysates were collected 7–10 days post transfection. Finally, the adenovirus was amplified by infecting 293 A cells with the crude lysate for 3–5 days, after which the adenovirus was purified using a Vivapure Adenopack 20 RT kit (Sartorius, #VS-AVPQ102). Ultimately, the titer of the ATF4-mCherry adenovirus was 1 × 10^10^ pfu/mL, the titer of the ZnT8-mCherry adenovirus was 4 × 10^10^ pfu/mL, and the titer of the control-mCherry adenovirus was 7 × 10^10^ pfu/mL.

### In vitro lineage-tracing strategy

SC-islet monolayer cells or human islets were infected with lentiviral vectors carrying either Rat INSx2 promoter–NLS-Cre-3×FLAG–PGK-Puro-WPRE or CMV-DIO-EGFP-WPRE. The culture medium was replaced the following day. After 48 h of infection, excessive zinc (3 μM ZnPTO or 200 μM ZnSO_4_) was added to the medium, and the cells were incubated for 24 h. The cells were then stained and subjected to confocal imaging. Lentiviruses were produced by OBiO Technology.

### Human islets used and treatments

Primary human islets were isolated at Navy Medical University according to the Clinical Islet Transplantation Consortium protocol (approval number: CHEC2018-111).^84,85^ In brief, pancreases from cadaveric donors were perfused with Liberase MTF C/T digestive enzyme (Roche, #05339880001) via the main pancreatic duct and then digested in a Ricordi chamber (Biorep). The digestion products were separated and purified using a continuous-density gradient of iodixanol, with densities ranging from 1.060 to 1.100 ± 0.01 g/mL. The isolated islets were then briefly placed on ice and transported to the laboratory, where they were immediately cultured in CMRL 1066 medium (Corning, #15-110-CV) supplemented with 2% human albumin, 1 mg/mL gabexate, and 0.6% penicillin/streptomycin.

### Chemicals and antibodies

The following chemicals were used: TSQ (Sigma‒Aldrich, #SML2656), ZnPTO (Sigma‒Aldrich, #H6377-10G), zinc sulfate heptahydrate (Sigma‒Aldrich, #Z0251), CuCl_2_ (Sinopharm Chemical Reagent Co., Ltd., #10007816), elesclomol (Selleck, #S1052), erastin (Selleck, #S7242), TPEN (Sigma–Aldrich, #P4413), and ISRIB (trans-isomer) (MedChemExpress, #HY-12495).

The following antibodies were used: anti-insulin (Sigma–Aldrich, #I2018, 1:400), anti-insulin (Boster, #BM0080, 1:100), anti-C-peptide (DSHB, #GN-ID4-S, 1:50), anti-C-peptide (Cell Signaling Technology, 4593S, 1:200), anti-ZnT8 (Thermo Fisher Scientific, #PA5-103252, 1:400), anti-ZnT8 (Lifespan Biosciences, #LS-C296473, 1:400), anti-Glucagon (Cell Signaling Technology, #2760S, 1:100), anti-ARX (R&D Systems, #AF-7068-SP, 1:50), anti-NKX6.1 (DSHB, #F55A12-c, 1:50), anti-GFP (MilliporeSigma, #06-896, 1:500), anti-mCherry (Abcam, #ab125096, 1:500), anti-CXCR4-PE (Thermo Fisher Scientific, #MHCXCR404, 1:100), anti-CD117-APC (Thermo Fisher Scientific, #CD11705, 1:100), anti-PDX1 (R&D Systems, #AF2419, 1:100), anti-somatostatin (Santa Cruz Biotechnology, #sc-7819, 1:100), anti-ATF4 (Proteintech, #10835-1-AP, 1:1000), anti-PERK (Cell Signaling Technology, #3192S, 1:1000), anti-p-PERK (ABclonal, #AP1501, 1:1000), anti-eIF2α (Beyotime, #AF6771, 1:1000), anti-p-eIF2α (Beyotime, #AF1237, 1:1000), anti-β-actin (Beyotime, #AF5001, 1:5000), anti-GAPDH (Beyotime, #AF1186, 1:5000), antibodies conjugated with Alexa Fluor 488 (715-545-150, 711-545-152, 712-545-150, 703-545-155, 1:500), Alexa Fluor 594 (715-585-150, 711-585-152, 712-585-153, 1:500), and Alexa Fluor 647 (715-605-151, 711-605-152, 712-605-153, 705-605-003, 713-605-147, 1:500) from Jackson ImmunoResearch, anti-mouse IgG HRP-linked antibody (Cell Signaling Technology, #7076P2, 1:10,000), and anti-rabbit IgG HRP-linked antibody (Cell Signaling Technology, #7074S, 1:10,000).

### Mouse studies

This study exclusively examined male mice. It is unknown whether the findings are applicable to female mice. *RIP-Cre*; *Rosa26*^*tdTomato*^ mice in the C57BL/6N background were kindly provided by Dr. Xin Xie. *Ins2-DreER*; *Rosa26-RSR-ZsGreen* mice were kindly provided by Dr. Bin Zhou. C57BL/6 and SCID-Beige mice were purchased from Beijing Vital River Laboratory Animal Technology Company and maintained in the animal facility of Tongji University, Shanghai, China according to protocols approved by the Biological Research Ethics Committee of Tongji University. Mice were housed in a controlled environment maintained at a constant temperature of 22  ±  1 °C with a 12-h light/dark cycle and 40%–60% humidity.

#### HFD-fed mouse model

*RIP-Cre*; *Rosa26*^*tdTomato*^ mice were fed an HFD for 6 months to establish the HFD-fed mouse model.

#### HFD-fed mouse model with zinc supplementation

To establish the HFD-fed mouse model with zinc supplementation, *RIP-Cre*; *Rosa26*^*tdTomato*^ mice were fed an HFD for 2 months and then fed an HFD supplemented with zinc in the drinking water (0.4 mg/mL) for 4 months.

#### Tamoxifen treatment

Six-week-old *Ins2-DreER*; *Rosa26-RSR-ZsGreen* male mice received intraperitoneal injections of tamoxifen dissolved in corn oil (200 mg/kg, 20 mg/mL; Beyotime, #ST1682-10 mL) once daily for three consecutive days. Control mice were injected with corn oil alone (10 μL/g; MedChemExpress, #HY-Y1888). β-cell labeling was analyzed one week after the final injection.

#### Transplantation into STZ-induced hyperglycemic mice

Hyperglycemic mice were induced by i.p. injection of 150 mg/kg streptozotocin (STZ) (YEASEN Biotechnology, #60256ES60) after 12 h of fasting. Random fed blood glucose levels were monitored using tail-blood samples, and mice with blood glucose levels greater than 16.7 mmol/L were selected for the study. Approximately 3 million SC-islets were transplanted under the kidney capsule after the mice were anesthetized with isoflurane. Nontransplanted diabetic mice were used as controls. Body weight and fed blood glucose levels were monitored weekly after transplantation.

#### Metabolic analysis

All metabolic analyses were performed on conscious, restrained mice. Blood glucose levels were monitored with a handheld glucometer using tail bleeds. For i.p. GTT, transplanted mice were fasted for 16 h. After i.p. injection of glucose (2 g/kg, 20% solution), blood glucose levels were measured at predetermined time points (0 min, 15 min, 30 min, 60 min, 90 min, and 120 min) using tail bleeds. For in vivo GSIS, transplanted mice were fasted for 16 h, and serum samples were collected from the eye socket before and 25 min after i.p. injection of glucose (2 g/kg, 20% solution).

### Quantitative real-time PCR

SC-islets were harvested by homogenization using a TianGen TRNzol Universal Kit (TianGen, #DP424) to isolate total RNA according to the manufacturer’s instructions. First-strand cDNA was synthesized using the Quantscript RT Kit (TianGen, #KR106) and used as a template for SYBR Green-based qPCR. β-Actin was used as a housekeeping control to normalize target gene expression, and the comparative threshold (ΔΔCt) method was used to quantify transcript abundance. The sequences of the qPCR primers used are provided in Supplementary information, Table S7.

### ChIP-qPCR

Cold DPBS was used to resuspend SC-β cells to 10^6^ cells/mL. Formaldehyde solution was then added to the cell suspension to reach a final concentration of 1%. After 10 min of rotation at RT, 125 mM glycine was added to terminate crosslinking and was accompanied by 5 min of rotation at RT. Following centrifugation at 2000 rpm for 5 min at 4 °C, the supernatant was discarded, and the cell pellet was stored at –80 °C for several weeks. A total of 10 μL of Dynabeads Protein A (Thermo Fisher Scientific, #10001D) was placed in a 1.5-mL tube, adsorbed on a magnet, and then washed with RIPA buffer 3 times. One microgram of anti-ATF4 antibody dissolved in 200 μL of RIPA buffer was added to the beads, followed by rotation at 4 °C for 3 h. Lysis buffer was added to the cell pellet until the volume reached 130 μL, followed by thorough mixing and vortexing on ice. The cell pellets were sonicated (peak power: 75, duty factor: 20, cycle: 200) and then transferred to a new tube and centrifuged at full speed for 20 min at 4 °C. After removal of the supernatant, the sample was diluted to 600 μL with RIPA buffer. A sample of 100–200 μL was added to the beads and rotated overnight at 4 °C, and 1/20 of the sample was reserved as the input sample. The next day, the samples were placed on a magnetic rack and washed twice with RIPA buffer and once with TE buffer (rotated for 5 min each time). Then, 200 μL of complete elution buffer containing proteinase K was added to each sample, and the samples were shaken at 68 °C and 1350 rpm for 2 h. After the samples had been decrosslinked, the supernatant was transferred into a new tube, and 200 μL of phenol, chloroform, 1/5 volume of sodium acetate, 1 µL of glycogen, and an equal volume of pre-cooled isopropanol were added in sequence and mixed thoroughly. The mixture was precipitated at –80 °C for 30 min, then centrifuged at maximum speed for 20 min at 4 °C. The sample was then washed twice with 70% ethanol. The DNA pellet was resuspended in 18 μL of ddH_2_O, and a concentration test was performed before qPCR.

### Flow cytometry

For intracellular staining, SC-islets were dissociated, fixed, permeabilized, and stained with primary antibodies for 30 min at RT. After two washes with PBS, the cells were incubated with fluorescence-conjugated secondary antibodies for 30 min at RT. Next, the cells were washed and resuspended in fluorescence-activated cell sorting (FACS) buffer (0.5% BSA in dPBS) for analysis on a BD FACSVerse instrument. Data were analyzed using FlowJo v10. For FACS, the *NKX6.1*^*mCherry/mCherry-*^*-INS*^*GFP/W*^ MEL1-derived SC-islets were dissociated into single cells with TrypLE and resuspended in FACS buffer. DNase I was added to eliminate DNA released from dead cells to prevent cell caking, and the tubes containing cells were placed on ice for preservation. SC-β cells were sorted on the basis of double-positive fluorescence for NKX6.1-mCherry and INS-GFP using a MoFlo Astrios (4 lasers). Pure SC-β cells were then aggregated into clusters using AggreWell 400 (STEMCell TECHNOLOGIES, #34415).

### Zinquin staining

SC-islets or human islets were washed with FACS buffer and stained with zinquin dye (1:600 dilution from a 20 mM stock, Sigma–Aldrich, #Z2251) for 20 min at 37 °C and 5% CO_2_. Zinquin-stained cells were washed with FACS buffer and observed immediately by microscopy with excitation at 361–367 nm and emmission at 482–488 nm.

### TSQ staining

Samples were incubated in PBST for 30 min for pretreatment. After draining, TSQ (50 μM, Sigma–Aldrich, SML2656) was added and incubated for 30 min. Samples were then washed three times with PBS for 5 min each. After drying, samples were mounted with antifade mounting medium (Beyotime, P0126).

### Western blot assay

SC-islets were harvested at the indicated time points and lysed directly in ice-cold RIPA buffer supplemented with a 1:100 dilution of protease inhibitor cocktail. Protein concentrations were quantified using a bicinchoninic acid protein assay kit (YEASEN, #20201ES76). Equal amounts of protein were mixed with loading buffer and loaded onto 10% SDS-PAGE gels for electrophoresis. Proteins were then transferred to nitrocellulose membranes (PALL, #66485). The membranes were blocked with 5% nonfat dry milk for 1 h and incubated with primary antibodies overnight at 4 °C. After 16 h, the membranes were washed and incubated with secondary antibodies for 1 h. The blots were visualized using enhanced chemiluminescence and imaged with an Amersham Imager 600.

### Trans-ISRIB treatment

#### Cell assay

For immunostaining of adherent SC-islets, cells were treated with 7 μM trans-ISRIB, with or without 3 μM ZnPTO added to the cell culture medium, for 24 h in an incubator (37 °C, 5% CO_2_). The cells were then fixed with 4% PFA and stained.

#### Mouse administration

The vehicle solution consisted of DPBS with 5% DMSO. Trans-ISRIB was administered at a dose of 2.5 mg/kg/day via i.p. injection to mice for 10 weeks after transplantation. The animals were randomly divided into vehicle and trans-ISRIB groups.

### Protein synthesis assay

Adherent SC-islets were plated at the proper density and cultured overnight. The next day, adherent SC-islets were treated with 25 nM ANS for 48 h. Protein synthesis was assessed using the Click-iT Plus OPP Protein Synthesis Assay Kit (Thermo Fisher, #C10456) according to the manufacturer’s instructions, and the cells were observed using an Olympus FV3000 microscope.

### Library construction and sequencing

Single-cell RNA-seq (scRNA-seq) libraries were prepared using the DNBelab C Series Single-Cell Library Prep Set V2.0 (MGI). The process included droplet encapsulation, emulsion breakage, reverse transcription, and amplification of cDNA and oligo products. Libraries were constructed with an input of 30,000 cells per condition, following the manufacturer’s instructions. Sequencing libraries were quantified using a Qubit ssDNA Assay Kit (Thermo Fisher, #Q10212) and sequenced on a DNBSEQ-T7 sequencer (MGI Tech) at JMDNA.

### Preprocessing of scRNA-seq data

Sequencing data were processed using the open-source DNBelab_C_Series_scRNA-analysis-software pipeline. Preprocessing was performed following standard procedures outlined in “Single-cell best practices”.^86^ Quality control was performed individually for glucose and glucose + zinc conditions using Omicverse.^87^ SoupX was used to remove ambient RNA contamination,^88^ and Scrublet was used for doublet removal.^89^ Expression matrices from CT and excessive zinc conditions were merged using the scVI method.^90^

Dimensionality reduction was performed using UMAP,^91^ and cell clusters were defined using the Leiden algorithm with a resolution of 0.3, yielding seven distinct clusters.^46^ Cell clusters were annotated using experimentally validated marker genes obtained from Scanpy results, including markers for α cells (GCG), β cells (INS), δ cells (SST), ε cells (GHRL), pancreatic progenitor cells (SOX9), and EC cells (FEV). DEGs between conditions were identified using the Wilcoxon test in Scanpy.

For human scRNA-seq data acquired from the Human Pancreas Analysis Program (HPAP; RRID: SCR_016202) database,^92^ we selected individuals with HbA1c levels ≥ 6.5 for patients with T2D (*n* = 13) and HbA1c levels ≤ 5.7 for ND individuals (*n* = 25) (Supplementary information, Table S1). Only endocrine cell types were retained and processed using the methods described above.

### GSEA

The resulting differential gene set was sorted on the basis of log fold changes. The sorted differential gene set was retrieved from the Molecular Signature Database (MSigDB), and Reactome_2024 pathways were selected.^93–95^ GSEA was performed using the prerank function in GSEApy, and pathways with a NOM *P* value ≤ 0.05 were considered to be enriched. The enriched pathways were then sorted on the basis of normalized enrichment score.

### Pseudotemporal ordering

For both the human scRNA-seq data and the SC-islet scRNA-seq data, pseudotime ordering was performed using the Monocle 3 package.^41–47^ Islet cells from both datasets were reordered, with cell clusters that showed high INS and low GCG expression used as the root node for construction of a transdifferentiation trajectory. The plot_cells function was used to visualize the trajectory in the reduced dimensional space. To examine the dynamics of gene expression during β-cell transdifferentiation, the plot_genes_in_pseudotime function was used to visualize the expression levels of marker genes along pseudotime.

### Statistics

Data were derived from at least three independent biological replicates. The data are presented as the means ± SEM. *P* values were calculated by two-tailed unpaired Student’s *t*-test if not otherwise indicated. For multiple comparisons, *P* values were calculated by one-way analysis of variance (ANOVA) or two-way repeated-measures ANOVA. *P  <  0.05, **P  <  0.01, ***P  <  0.001, ****P <  0.0001; ns, not significant.

## Supplementary information

Supplementary information, Figure 1

Supplementary information, Figure 2

Supplementary information, Figure 3

Supplementary information, Figure 4

Supplementary information, Figure 5

Supplementary information, Figure 6

Supplementary information, Figure 7

Supplementary information, Figure 8

Supplementary information, Figure 9

Supplementary information, Figure 10

Supplementary information, Figure 11

Supplementary information, Figure 12

Supplementary information, Figure 13

Supplementary information, Figure 14

Supplementary information, Figure 15

Supplementary information, Figure 16

Supplementary information, Figure 17

Supplementary information, Figure 18

Supplementary information, Figure 19

Supplementary information, Figure 20

Supplementary information, Table S1

Supplementary information, Table S2

Supplementary information, Table S3

Supplementary information, Table S4

Supplementary information, Table S5

Supplementary information, Table S6

Supplementary information, Table S7

## Data Availability

All relevant data are reported in the main text or Supplementary information. The data supporting the findings of this study are available from the corresponding authors on request. The single-cell RNA-seq data are available at https://www.ncbi.nlm.nih.gov/geo/query/acc.cgi?acc=GSE236316.
