# Supplementary material for: Zinc accumulation-induced integrated stress response triggers β-cell identity loss
Source: Cell Res. 2026 Jan 28;36(5):359–76. doi: 10.1038/s41422-026-01222-y (PMC13092640; doi:10.1038/s41422-026-01222-y)
Supplement: Supplementary file 1 — Supplementary information, Figure 1 [file 41422_2026_1222_MOESM1_ESM.pdf]

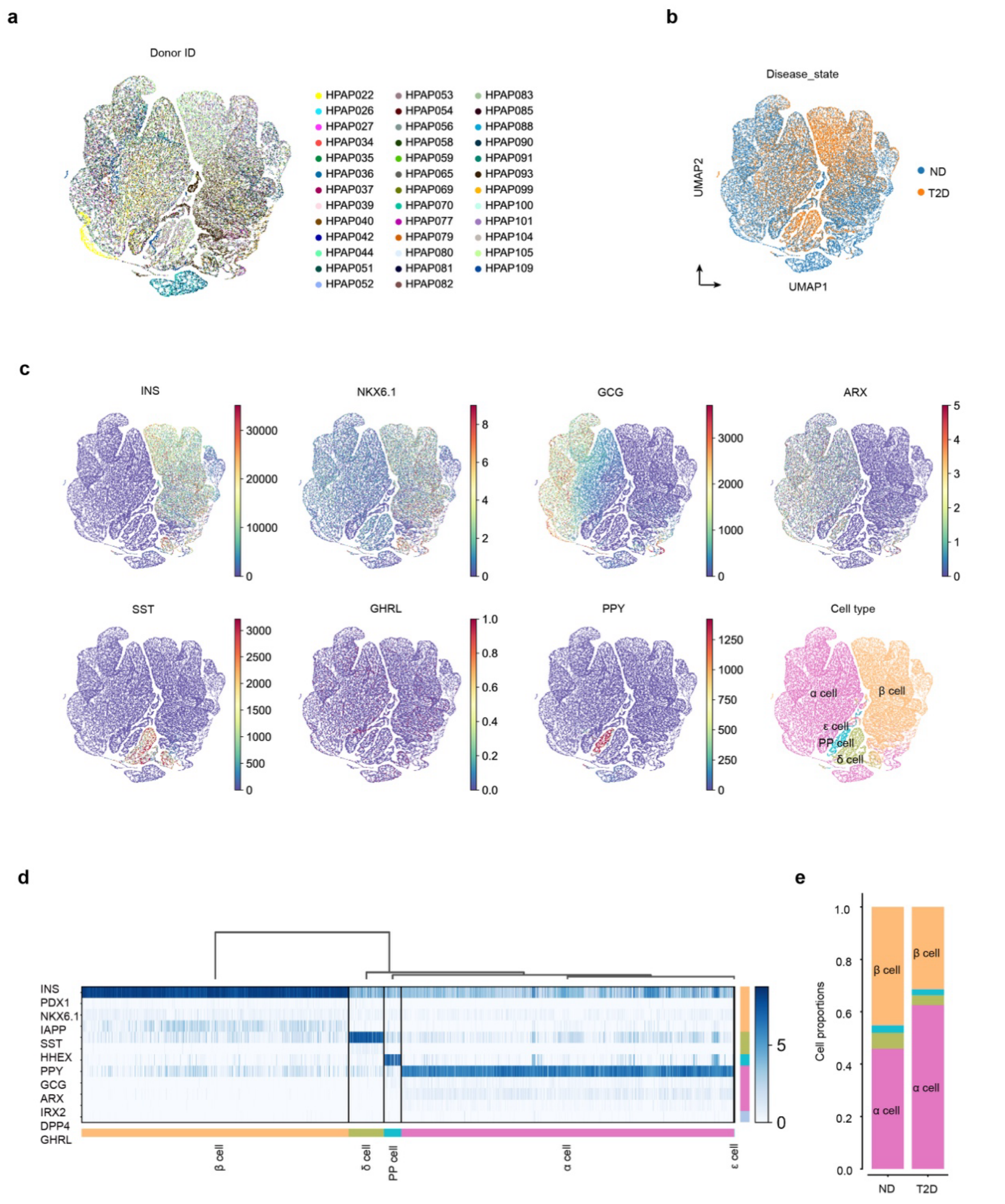

Supplementary information, Figure S1 Single-cell analysis of cellular composition and gene expression in human islets. a UMAP plot showing the distribution of human pancreatic islet cells from individual donors, with clusters colored by donor ID. **b** UMAP plot showing the distribution of human pancreatic islet samples according to disease state. **c** UMAP plots depicting the expression of pancreatic endocrine cell type marker genes. **d** Heatmap of gene expression in pancreatic endocrine cell types. **e** Proportions of different pancreatic endocrine cell types in human islets across disease states.
