# Supplementary material for: Zinc accumulation-induced integrated stress response triggers β-cell identity loss
Source: Cell Res. 2026 Jan 28;36(5):359–76. doi: 10.1038/s41422-026-01222-y (PMC13092640; doi:10.1038/s41422-026-01222-y)
Supplement: Supplementary file 2 — Supplementary information, Figure 2 [file 41422_2026_1222_MOESM2_ESM.pdf]

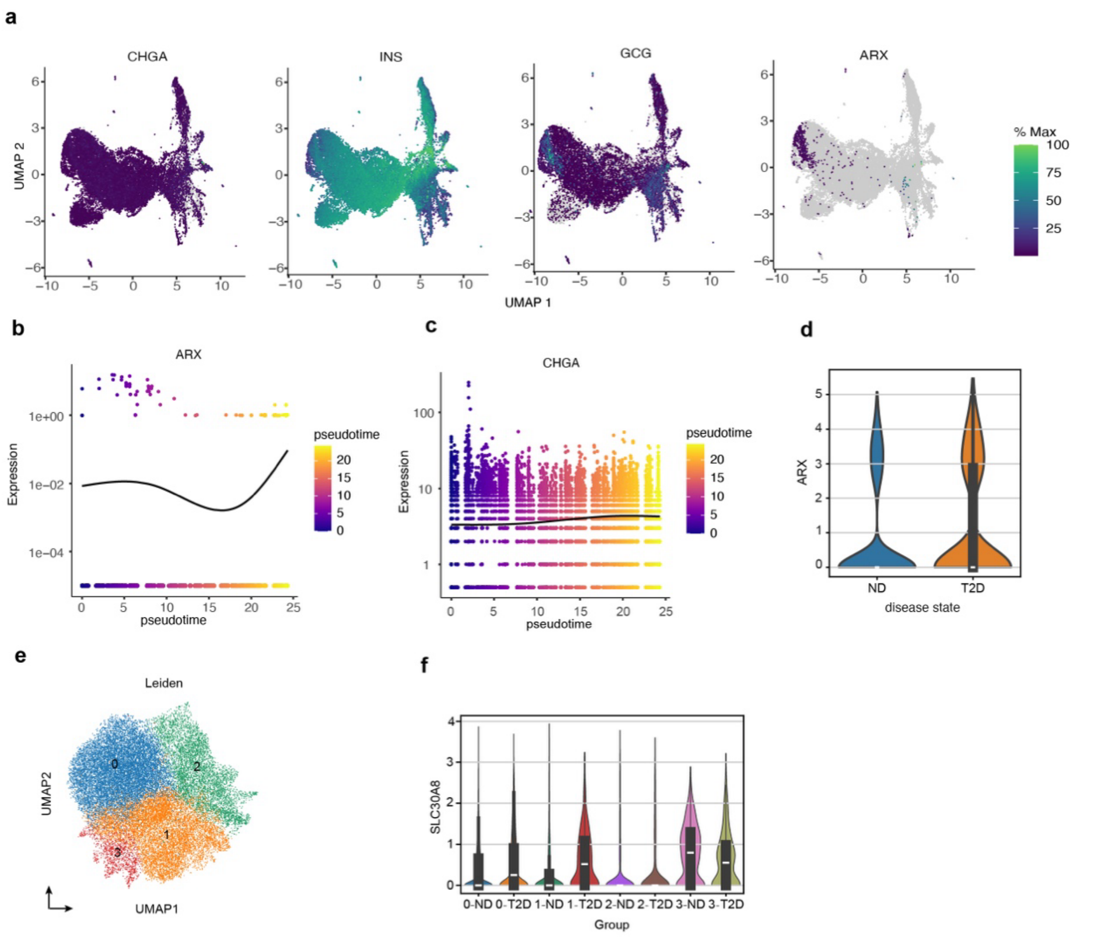

Supplementary information, Figure S2 Single-cell analysis of gene expression dynamics in human islets. a UMAP plots showing the expression of *CHGA*, *INS*, *GCG* and *ARX* across pancreatic β cells along pseudotime. **b, c** The expression dynamics of *ARX* (**b**) and *CHGA* (**c**) along pseudotime. **d** Violin plot showing the expression of ARX in β cells. **e** UMAP plot categorizing β cell subclusters. **f** Violin plot showing changes in *SLC30A8* expression across β cell subclusters between ND donors and patients with T2D.
