# Supplementary material for: Zinc accumulation-induced integrated stress response triggers β-cell identity loss
Source: Cell Res. 2026 Jan 28;36(5):359–76. doi: 10.1038/s41422-026-01222-y (PMC13092640; doi:10.1038/s41422-026-01222-y)
Supplement: Supplementary file 3 — Supplementary information, Figure 3 [file 41422_2026_1222_MOESM3_ESM.pdf]

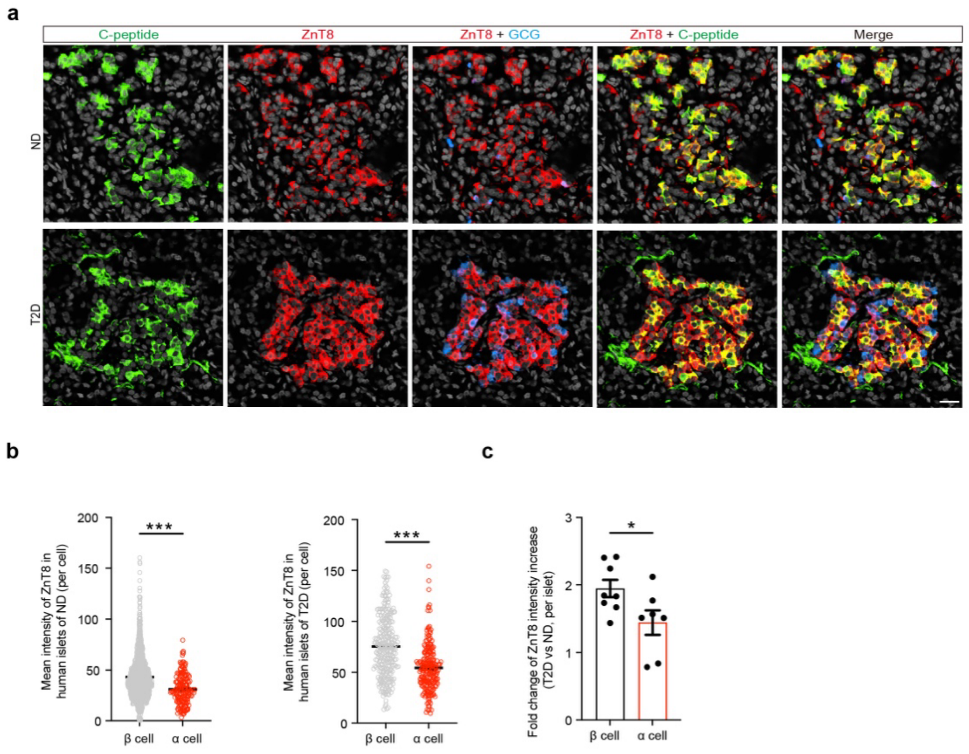

Supplementary information, Figure S3 β cells exhibits elevated expression levels of ZnT8 in patients with T2D. a Representative immunofluorescent images for C-peptide (green), ZnT8 (red) and GCG (blue) in human primary islets from ND and patients with T2D. Scale bar, 25 μm. **b** Mean intensity measurements of ZnT8 in human primary β cells and α cells from ND (β cells, n = 1113; α cells, n = 181) and patients with T2D (β cells, n = 283; α cells, n = 231). **c** Fold change (patients with T2D vs ND) of average ZnT8 intensity in human primary β cells (per islet, n = 8) and α cells (per islet, n = 7). Unpaired two-tailed *t* test was used to analyze for **b, c** $*p < 0.05$, $**p < 0.01$, $***p < 0.001$. Data are presented as mean ± s.e.m. Individual data points are shown for all bar graphs.
