# Supplementary material for: Zinc accumulation-induced integrated stress response triggers β-cell identity loss
Source: Cell Res. 2026 Jan 28;36(5):359–76. doi: 10.1038/s41422-026-01222-y (PMC13092640; doi:10.1038/s41422-026-01222-y)
Supplement: Supplementary file 4 — Supplementary information, Figure 4 [file 41422_2026_1222_MOESM4_ESM.pdf]

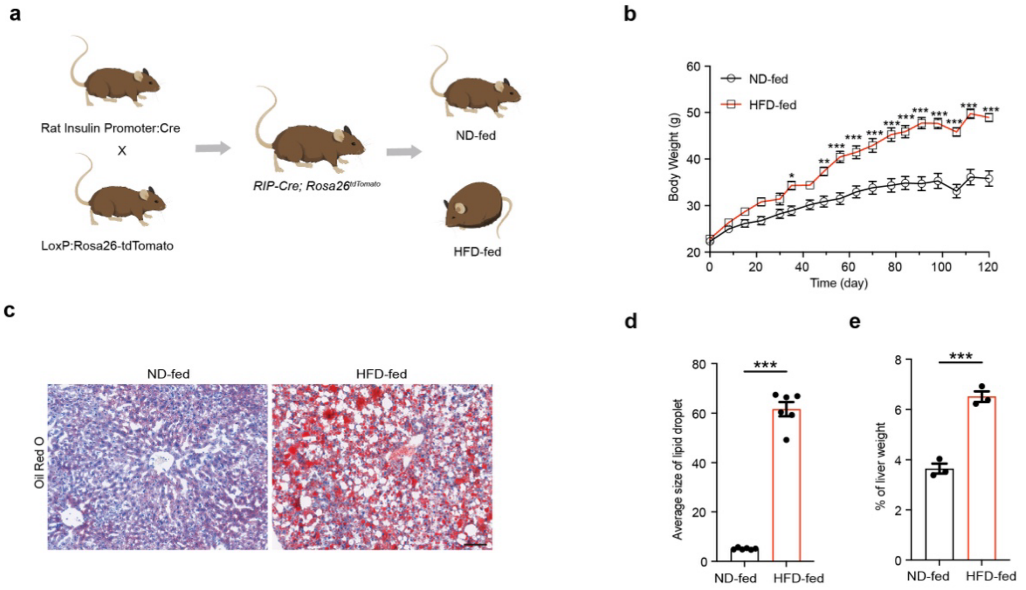

Supplementary information, Figure S4 Establishment of HFD-induced diabetic mouse model using *RIP-Cre; Rosa26^{tdTomato}* mice. a Schematic diagram illustrating the generation of *RIP-Cre; Rosa26^{tdTomato}* mice by crossing mice carrying the *Rat insulin promoter: Cre* transgene with those carrying the *LoxP: Rosa26^{tdTomato}* allele, followed by HFD feeding for 6 months. The diagram is created by figdraw.com. **b** Body weight of the ND-fed mice and HFD-fed mice. n = 5. **c, d** Representative Oil Red O staining images of liver sections (**c**) and quantification of the average lipid droplet size (**d**) in ND-fed and HFD-fed mice. n = 6. Scale bar, 100 μm. **e**, Percentage of liver weight relative to body weight in ND-fed and HFD-fed mice. n = 3. Two-way ANOVA with Sidak's multiple-comparisons was used to analyze for **b**. Unpaired two-tailed *t* test was used to analyze for **d** and **e**. *$p < 0.05$, **$p < 0.01$, ***$p < 0.001$. Data are presented as mean ± s.e.m. Individual data points are shown for all bar graphs.
