# Supplementary material for: Zinc accumulation-induced integrated stress response triggers β-cell identity loss
Source: Cell Res. 2026 Jan 28;36(5):359–76. doi: 10.1038/s41422-026-01222-y (PMC13092640; doi:10.1038/s41422-026-01222-y)
Supplement: Supplementary file 5 — Supplementary information, Figure 5 [file 41422_2026_1222_MOESM5_ESM.pdf]

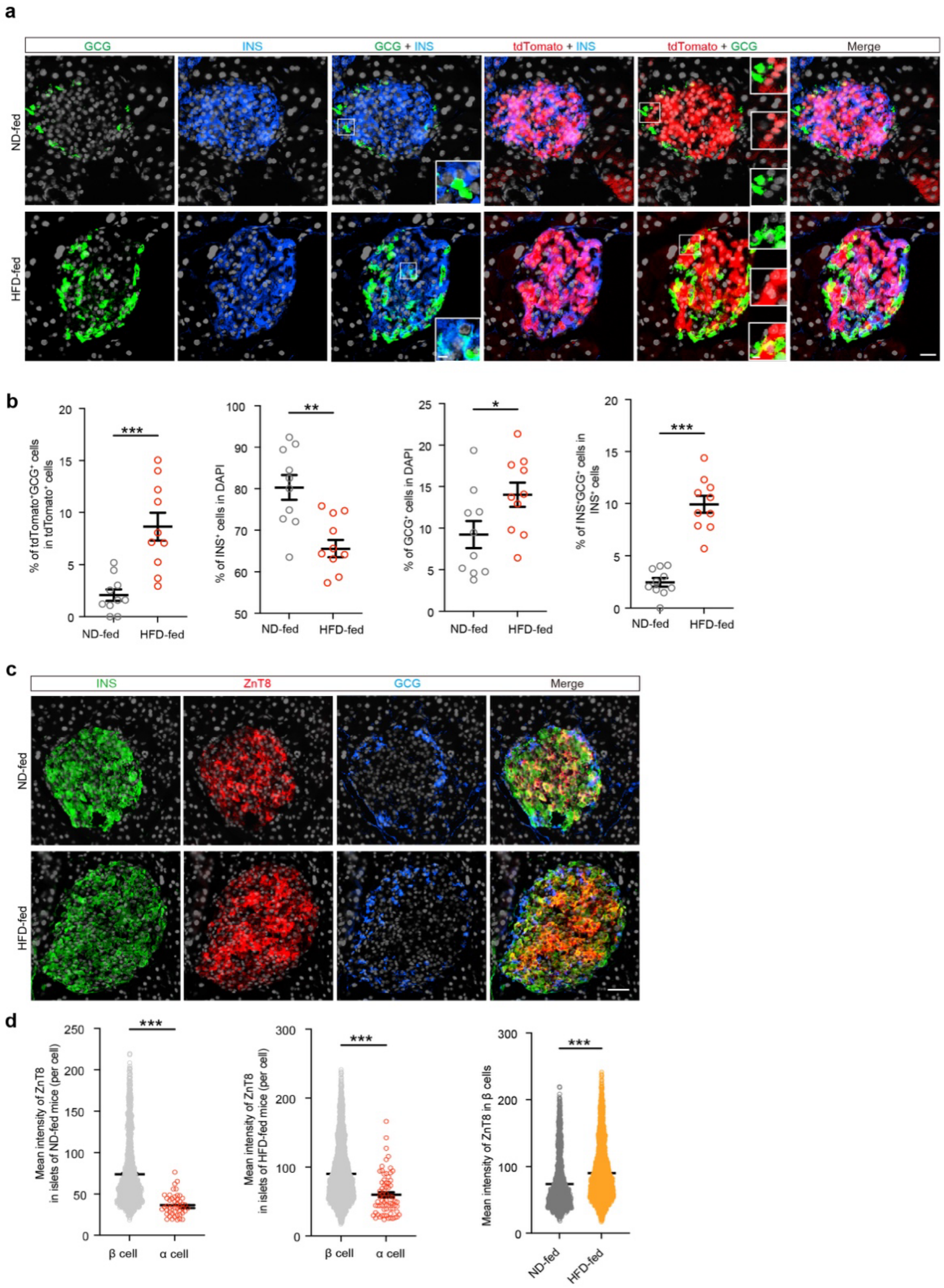

Supplementary information, Figure S5 Pancreatic β cell undergoes identity loss accompanied by zinc accumulation in HFD induced diabetic mouse model. a, b Representative immunofluorescence images (**a**) and quantification (**b**) showing the percentages of INS⁺ cells (blue) and GCG⁺ cells (green) among the total number of DAPI⁺ cells (grey), as well as the proportion of bi-hormonal INS⁺GCG⁺ cells among the total INS⁺ cells, and the proportion of GCG⁺tdTomato⁺ cells among the total tdTomato⁺ cells (red) in islets from ND-fed mice and HFD-fed mice. n = 10. Scale bar in low magnification, 25 μm; Scale bar in high magnification, 5 μm. **c, d** Representative immunofluorescent images (**c**) and the quantification (**d**) for mean intensity of ZnT8 in mouse primary α cells and β cells from ND-fed (β cells, n = 1248; α

cells, n = 47) and HFD-fed mice (β cells, n = 2766; α cells, n = 69). Scale bar, 50 μm. Unpaired two-tailed *t* test was used to analyze for **b, d**. *$p < 0.05$, **$p < 0.01$, ***$p < 0.001$. Data are presented as mean ± s.e.m.
