# Supplementary material for: Zinc accumulation-induced integrated stress response triggers β-cell identity loss
Source: Cell Res. 2026 Jan 28;36(5):359–76. doi: 10.1038/s41422-026-01222-y (PMC13092640; doi:10.1038/s41422-026-01222-y)
Supplement: Supplementary file 6 — Supplementary information, Figure 6 [file 41422_2026_1222_MOESM6_ESM.pdf]

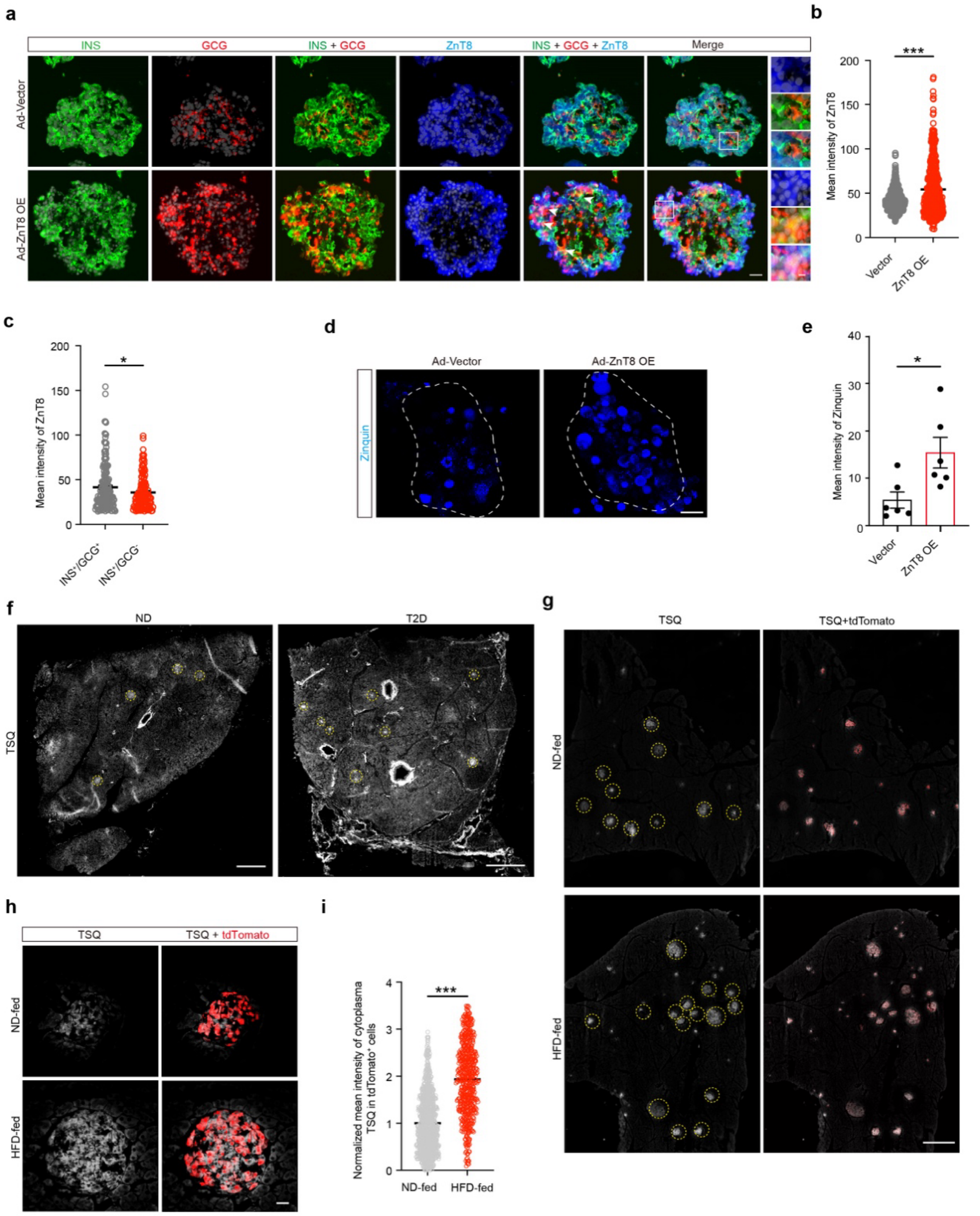

Supplementary information, Figure S6 Zinc is accumulated in diabetic islets. a-c Representative immunofluorescent images (**a**) and quantification (**b**) for mean intensity of ZnT8 in human islets infected with adenovirus carrying either an empty vector (n = 1072 cells) or ZnT8 OE construct (n = 440 cells) under high glucose (33 mM) medium, as well as quantification (**c**) for mean intensity of ZnT8 in INS^{+}GCG^{+} cells (n = 174 cells) and INS^{+}GCG^{-} cells (n = 132 cells) in the group of ZnT8 OE human islets. Scale bar in high magnification, 5 μm; Scale bar in low magnification, 25 μm. **d, e** Zinquin staining (**d**) and quantification of mean fluorescence intensity (**e**) in human primary islets infected with adenovirus carrying either an empty vector or ZnT8 OE construct. n = 6. Scale bar, 25 μm. **f**

Representative low-magnification images of TSQ staining in human pancreatic sections from ND and patients with T2D. Scale bar, 1 mm. **g** Representative low-magnification images of TSQ staining in pancreatic sections from ND-fed mice and HFD-fed mice. Scale bar, 0.8 mm. **h, i** Representative TSQ staining images (**h)** and normalized mean intensity measurements (**i**) for the $tdTomato^+$ cells of islets from ND-fed mice (n = 1032 cells) or HFD-fed mice (n = 596 cells). Scale bar, 25 μm. Unpaired two-tailed *t* test was used to analyze for **b, c, e** and **i**. *$p < 0.05$, **$p < 0.01$, ***$p < 0.001$. Data are presented as mean ± s.e.m. Individual data points are shown for all bar graphs.
