# Supplementary material for: Zinc accumulation-induced integrated stress response triggers β-cell identity loss
Source: Cell Res. 2026 Jan 28;36(5):359–76. doi: 10.1038/s41422-026-01222-y (PMC13092640; doi:10.1038/s41422-026-01222-y)
Supplement: Supplementary file 7 — Supplementary information, Figure 7 [file 41422_2026_1222_MOESM7_ESM.pdf]

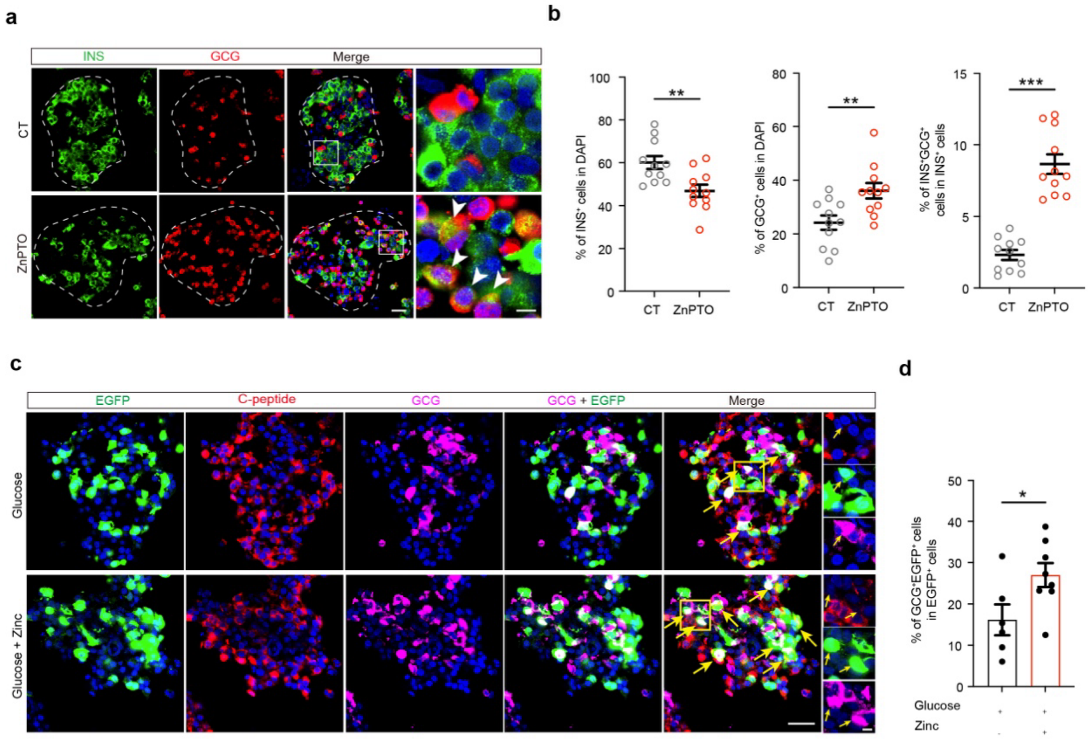

Supplementary information, Figure S7 Accumulated Zinc leads to identity loss of human primary β cells. a, b Representative immunofluorescent images (**a**) and quantification (**b**) for the percentages of INS$^+$ cells (green) and GCG$^+$ cells (red) among the total number of DAPI$^+$ cells, as well as the proportion of bi-hormonal INS$^+$GCG$^+$ cells among total INS$^+$ cells in human islets with or without 3 μM ZnPTO treatment for 24 h. n = 11. Scale bar in high magnification, 5 μm; Scale bar in low magnification, 25 μm. **c, d** Representative immunofluorescent images (**c**) and the quantification for the percentages (**d**) of GCG$^+$EGFP$^+$ cells in the total EGFP$^+$ cells from human islets infected with *RIP-Cre* and *CMV-DIO-EGFP* lentiviral vectors under high glucose with (n = 8) or without zinc treatment (n = 6). Scale bar in high magnification, 5 μm; Scale bar in low magnification, 25 μm. Unpaired two-tailed *t* test was used to analyze for **b,** and **d**. $*p < 0.05$, $**p < 0.01$, $***p < 0.001$. Data are presented as mean ± s.e.m. Individual data points are shown for all bar graphs.
