# Supplementary material for: Zinc accumulation-induced integrated stress response triggers β-cell identity loss
Source: Cell Res. 2026 Jan 28;36(5):359–76. doi: 10.1038/s41422-026-01222-y (PMC13092640; doi:10.1038/s41422-026-01222-y)
Supplement: Supplementary file 8 — Supplementary information, Figure 8 [file 41422_2026_1222_MOESM8_ESM.pdf]

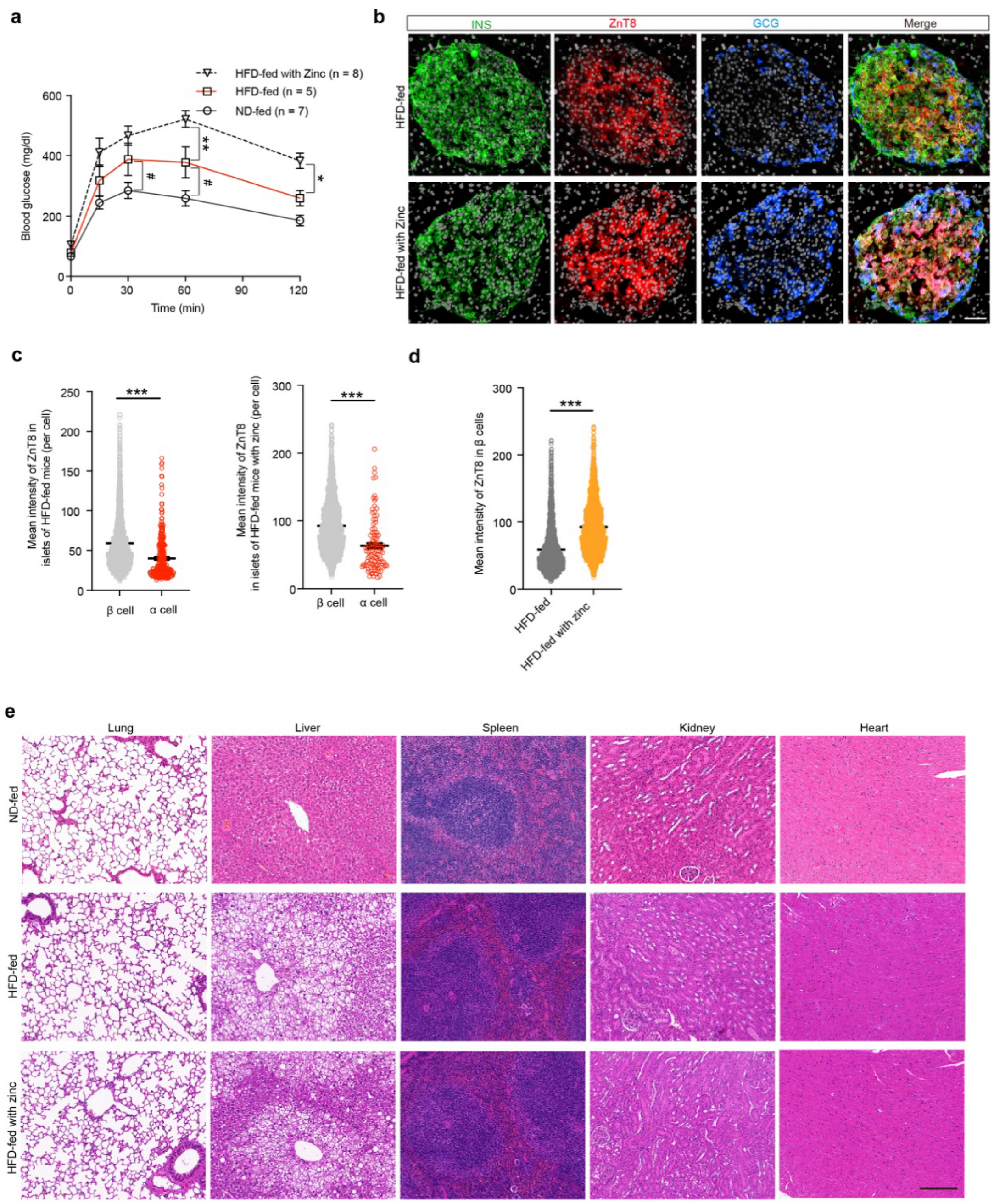

Supplementary information, Figure S8 Additional analysis of β cell identity loss in HFD-fed mice and HFD-fed mice with zinc supplementation. a i.p. GTT in ND-fed mice (black line: ND-fed, n = 7), HFD-fed mice (red line: HFD-fed, n = 5) and HFD-fed mice with zinc supplementation (black dotted line: HFD-fed with zinc, n = 8). **b-d** Representative immunofluorescent images (**b**) and the quantification (**c, d**) for mean intensity of ZnT8 in mouse α cells and β cells from HFD-fed (β cells, n = 2078; α cells, n = 297) and HFD-fed mice with zinc supplementation (β cells, n = 2203; α cells, n = 106). Scale bar, 50 μm. **e** Representative H&E staining images of major organs from ND-fed mice, HFD-fed mice and HFD-fed mice with zinc supplementation, respectively. Scale bar, 200 μm. Two-way ANOVA with Dunnett's multiple-comparisons was used to analyze for **a**. Unpaired two-tailed *t* test was used to analyze for **c** and **d**. *$p < 0.05$, **$p < 0.01$, ***$p < 0.001$. Data are presented as mean ± s.e.m.
