# Supplementary material for: Zinc accumulation-induced integrated stress response triggers β-cell identity loss
Source: Cell Res. 2026 Jan 28;36(5):359–76. doi: 10.1038/s41422-026-01222-y (PMC13092640; doi:10.1038/s41422-026-01222-y)
Supplement: Supplementary file 9 — Supplementary information, Figure 9 [file 41422_2026_1222_MOESM9_ESM.pdf]

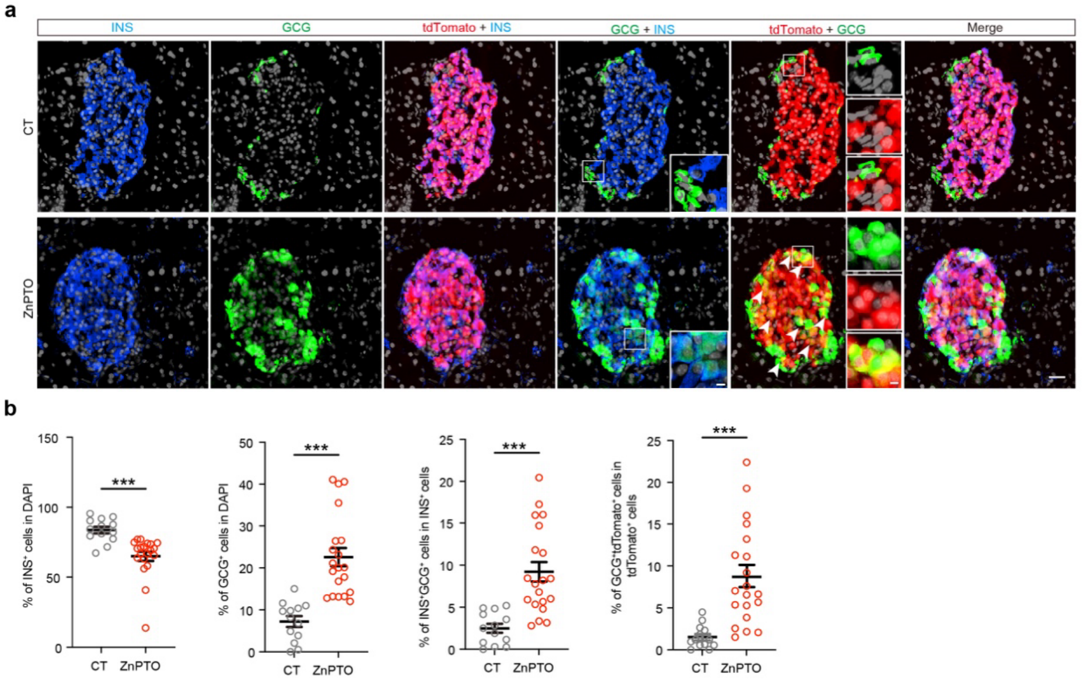

Supplementary information, Figure S9 In situ pancreatic injection of ZnPTO induces β cell identity loss in *RIP-Cre; Rosa26*^{tdTomato} mouse islets. a, b Representative immunofluorescence images (**a**) and quantification (**b**) showing the percentages of INS$^+$ cells (blue) and GCG$^+$ cells (green) among the total number of DAPI$^+$ cells (grey), as well as the proportion of bi-hormonal INS$^+$GCG$^+$ cells among total INS$^+$ cells, and the proportion of GCG$^+$tdTomato$^+$ cells among total tdTomato$^+$ cells (red) in islets from CT (n = 13) or ZnPTO injected mice (n = 20). Scale bar in low magnification, 25 μm; Scale bar in high magnification, 5 μm. Unpaired two-tailed *t* test was used to analyze for **b**. *$p < 0.05$, **$p < 0.01$, ***$p < 0.001$. Data are presented as mean ± s.e.m.
