# Supplementary material for: Zinc accumulation-induced integrated stress response triggers β-cell identity loss
Source: Cell Res. 2026 Jan 28;36(5):359–76. doi: 10.1038/s41422-026-01222-y (PMC13092640; doi:10.1038/s41422-026-01222-y)
Supplement: Supplementary file 10 — Supplementary information, Figure 10 [file 41422_2026_1222_MOESM10_ESM.pdf]

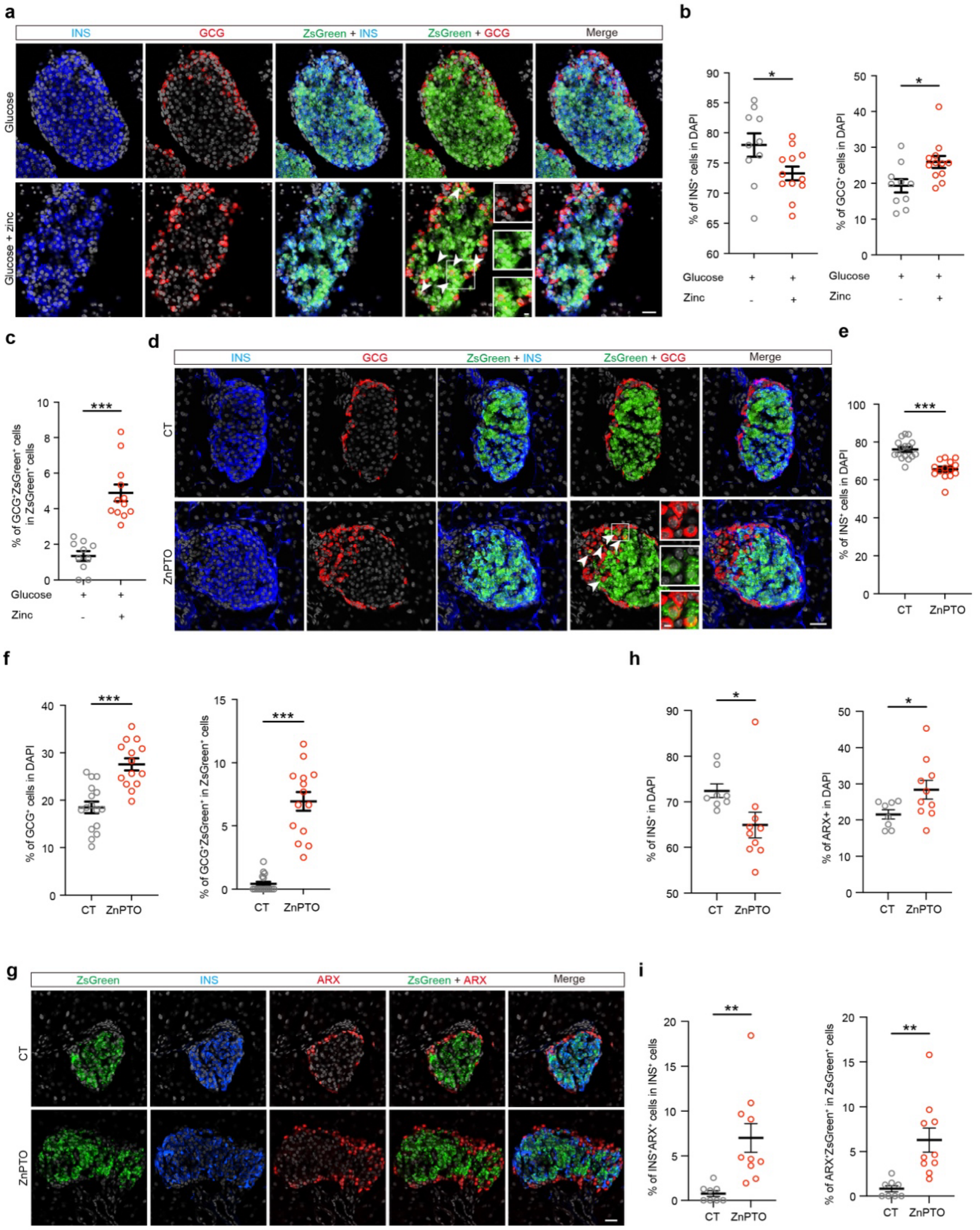

Supplementary information, Figure S10 Zinc accumulation induces β cell identity loss in *Ins2-DreER; Rosa26-RSR-ZsGreen* mouse islets. a-c Representative immunofluorescent images (**a**) and the quantification (**b, c**) for the percentages of INS^+ cells (blue) and GCG^+ cells (red) among the total number of $DAPI^+$ cells (grey), as well as the ratio of $GCG^+ZsGreen^+$ cells to the total $ZsGreen^+$ cells in mouse islets with (n = 12) or without (n = 10) excessive zinc (200 μM ZnSO4) treatment under high glucose (33 mM) environment. White arrows indicating $GCG^+ZsGreen^+$ cells. Scale bar in low magnification, 25 μm; Scale bar in high magnification, 5 μm. **d-f** Representative immunofluorescent images (**d**) and the quantification (**e, f**) showing the percentages of INS^+ cells (blue) and GCG^+ cells (red) among the total

number of DAPI$^+$ cells (grey), and the proportion of GCG$^+$ZsGreen$^+$ cells to the total ZsGreen$^+$ cells in mouse islets from CT (n = 16) or ZnPTO injected mice (n = 14). Scale bar in low magnification, 25 μm; Scale bar in high magnification, 5 μm. **g-i** Representative immunofluorescent images (**g**) and the quantification (**h, i**) showing the percentages of INS$^+$ cells (blue) and ARX$^+$ cells (red) among the total number of DAPI$^+$ cells (grey), as well as the proportion of bi-hormonal INS$^+$ARX$^+$ cells among total INS$^+$ cells, and the proportion of ARX$^+$ZsGreen$^+$ cells to the total ZsGreen$^+$ cells in mouse islets from CT (n = 8) or ZnPTO injected mice (n = 10). Scale bar, 25 μm. Unpaired two-tailed *t* test was used to analyze in this figure. *$p < 0.05$, **$p < 0.01$, ***$p < 0.001$. Data are presented as mean ± s.e.m.
