# Supplementary material for: Zinc accumulation-induced integrated stress response triggers β-cell identity loss
Source: Cell Res. 2026 Jan 28;36(5):359–76. doi: 10.1038/s41422-026-01222-y (PMC13092640; doi:10.1038/s41422-026-01222-y)
Supplement: Supplementary file 11 — Supplementary information, Figure 11 [file 41422_2026_1222_MOESM11_ESM.pdf]

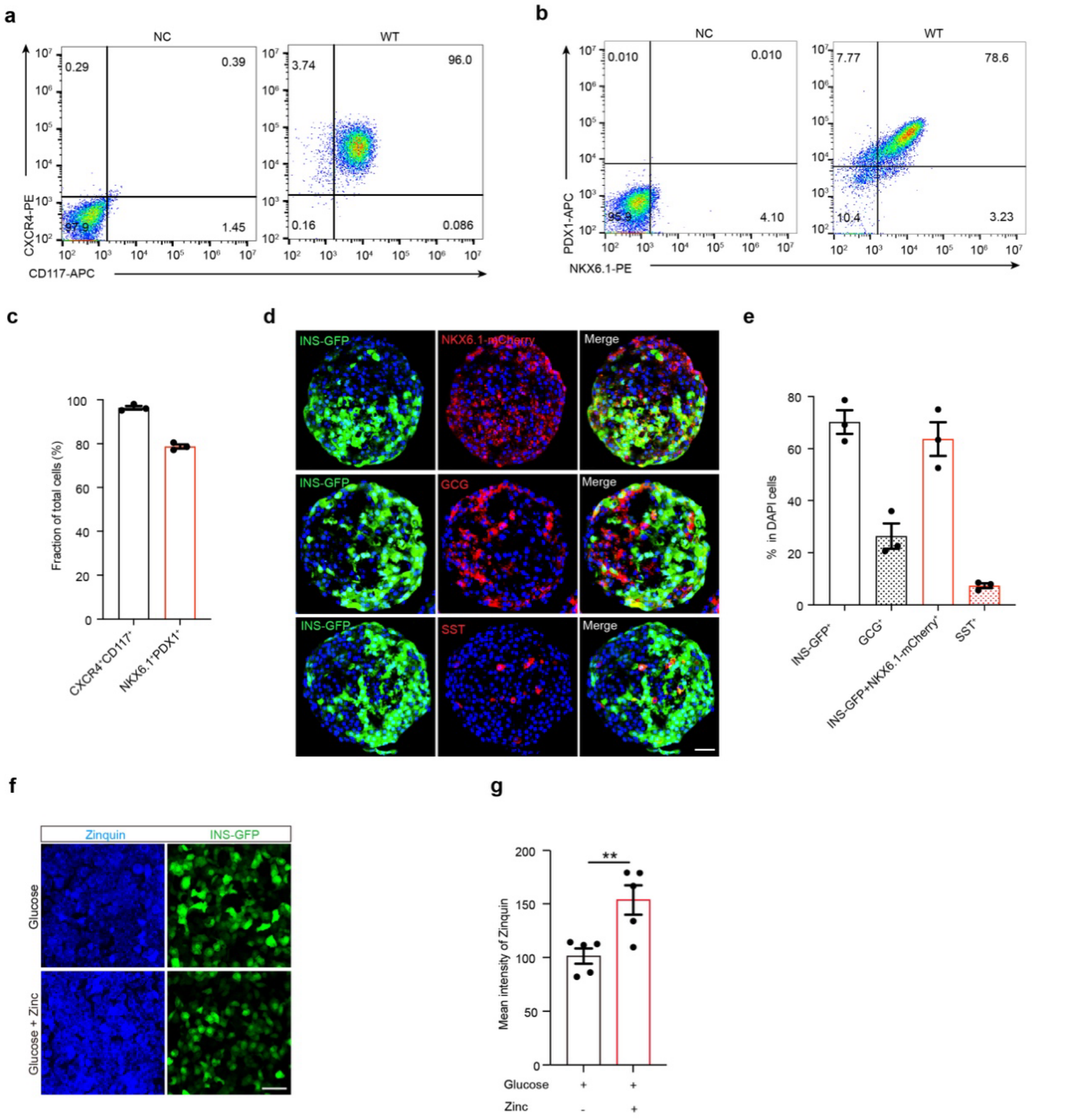

Supplementary information, Figure S11 Pancreatic β cell differentiation efficiency towards SC-islets. a, b Representative FACS plots showing the percentages of CD117$^+$CXCR4$^+$ in definitive endoderm cells (**a**) and PDX1$^+$NKX6.1$^+$ in pancreatic progenitor cells (**b**). **c** FACS quantifications of CXCR4$^+$CD117$^+$ and NKX6.1$^+$PDX1$^+$ in WT SC-islets. n = 3. **d, e** Representative immunofluorescent images (**d**) and quantification (**e**) for percentages of INS-GFP$^+$, GCG$^+$, INS-GFP$^+$NKX6.1-mCherry$^+$ and SST$^+$ cells among the total number of DAPI$^+$ cells from mature SC-islets. n = 3. Scale bar, 50 μm. **f, g** Zinquin staining (**f**) and quantification of mean fluorescence intensity (**g**) in adherent SC-islets under high glucose with or without zinc treatment. n = 5. Scale bar, 50 μm. Unpaired two-tailed *t* test was used to analyze for **g**. *$p < 0.05$, **$p < 0.01$, ***$p < 0.001$. Data are presented as mean ± s.e.m. Individual data points are shown for all bar graphs.
