# Supplementary material for: Zinc accumulation-induced integrated stress response triggers β-cell identity loss
Source: Cell Res. 2026 Jan 28;36(5):359–76. doi: 10.1038/s41422-026-01222-y (PMC13092640; doi:10.1038/s41422-026-01222-y)
Supplement: Supplementary file 12 — Supplementary information, Figure 12 [file 41422_2026_1222_MOESM12_ESM.pdf]

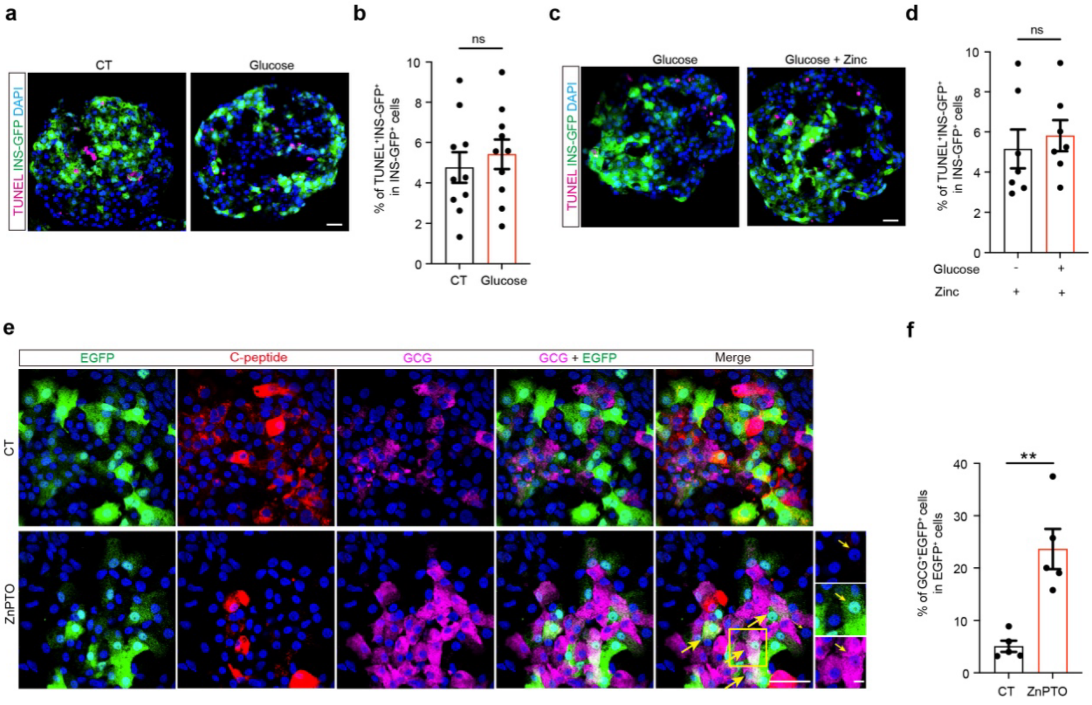

Supplementary information, Figure S12 Additional analysis for SC-β cell identity loss under excessive zinc. a, b Representative immunofluorescent images (**a**) and the quantification (**b**) for the percentages of $TUNEL^{+}INS\text{-}GFP^{+}$ cells in total $INS\text{-}GFP^{+}$ cells from SC-islets with or without high glucose. n = 10. Scale bar, 25 μm. **c, d** Representative immunofluorescent images (**c**) and the quantification (**d**) for percentages of $TUNEL^{+}INS\text{-}GFP^{+}$ cells in total $INS\text{-}GFP^{+}$ cells from SC-islets treated with or without excessive zinc under high glucose. n = 7. Scale bar, 25 μm. **e**, **f** Representative immunofluorescent images (**e**) and the quantification for the percentages (**f**) of $GCG^{+}EGFP^{+}$ cells in the total $EGFP^{+}$ cells from SC-islets infected with *RIP-Cre* and *CMV-DIO-EGFP* lentiviral vectors with or without ZnPTO treatment. n = 5. Scale bar in high magnification, 5 μm; Scale bar in low magnification, 25 μm.Unpaired two-tailed *t* test was used to analyze for this figure. *$p < 0.05$, **$p < 0.01$, ***$p < 0.001$, ns, no significance. Data are presented as mean ± s.e.m. Individual data points are shown for all bar graphs.
