# Supplementary material for: Zinc accumulation-induced integrated stress response triggers β-cell identity loss
Source: Cell Res. 2026 Jan 28;36(5):359–76. doi: 10.1038/s41422-026-01222-y (PMC13092640; doi:10.1038/s41422-026-01222-y)
Supplement: Supplementary file 13 — Supplementary information, Figure 13 [file 41422_2026_1222_MOESM13_ESM.pdf]

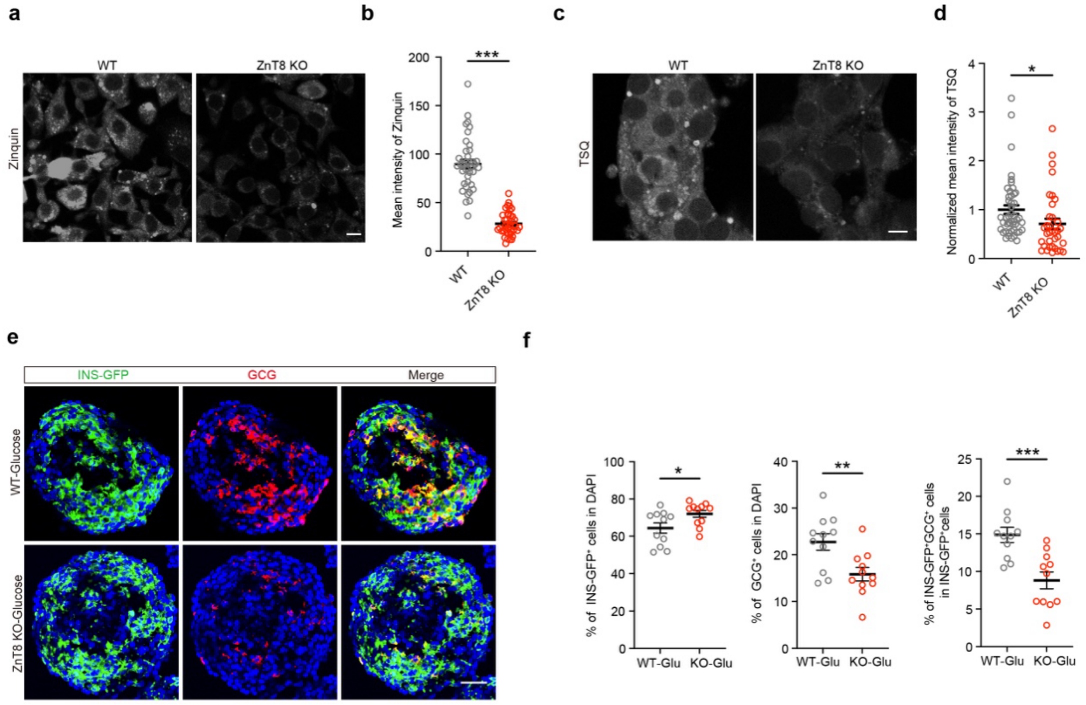

Supplementary information, Figure S13 ZnT8 KO protects SC-β cell identity loss under high glucose environment. a, b Representative Zinquin staining images (**a**) and mean intensity measurements (**b**) in WT and ZnT8 KO adherent SC-islets. n = 40. Scale bar, 5 µm. **c, d** Representative TSQ staining images (**c**) and normalized mean intensity measurements (**d**) in WT (n = 47) and ZnT8 KO adherent SC-islets (n = 35). Scale bar, 5 µm. **e, f** Representative immunofluorescent images (**e**) and quantification (**f**) for percentages of INS-GFP^{+} cells (green), GCG^{+} cells (red) among the total number of DAPI (blue), as well as the proportion of bi-hormonal INS-GFP^{+}GCG^{+} cells among the total INS-GFP^{+} cells in the WT and ZnT8 KO SC-islets under high glucose treatment. n = 11. Scale bar, 50 µm. Unpaired two-tailed *t* test was used to analyze in this figure. *$p < 0.05$, **$p < 0.01$, ***$p < 0.001$. Data are presented as mean ± s.e.m. Individual data points are shown for all bar graphs.
