# Supplementary material for: Zinc accumulation-induced integrated stress response triggers β-cell identity loss
Source: Cell Res. 2026 Jan 28;36(5):359–76. doi: 10.1038/s41422-026-01222-y (PMC13092640; doi:10.1038/s41422-026-01222-y)
Supplement: Supplementary file 14 — Supplementary information, Figure 14 [file 41422_2026_1222_MOESM14_ESM.pdf]

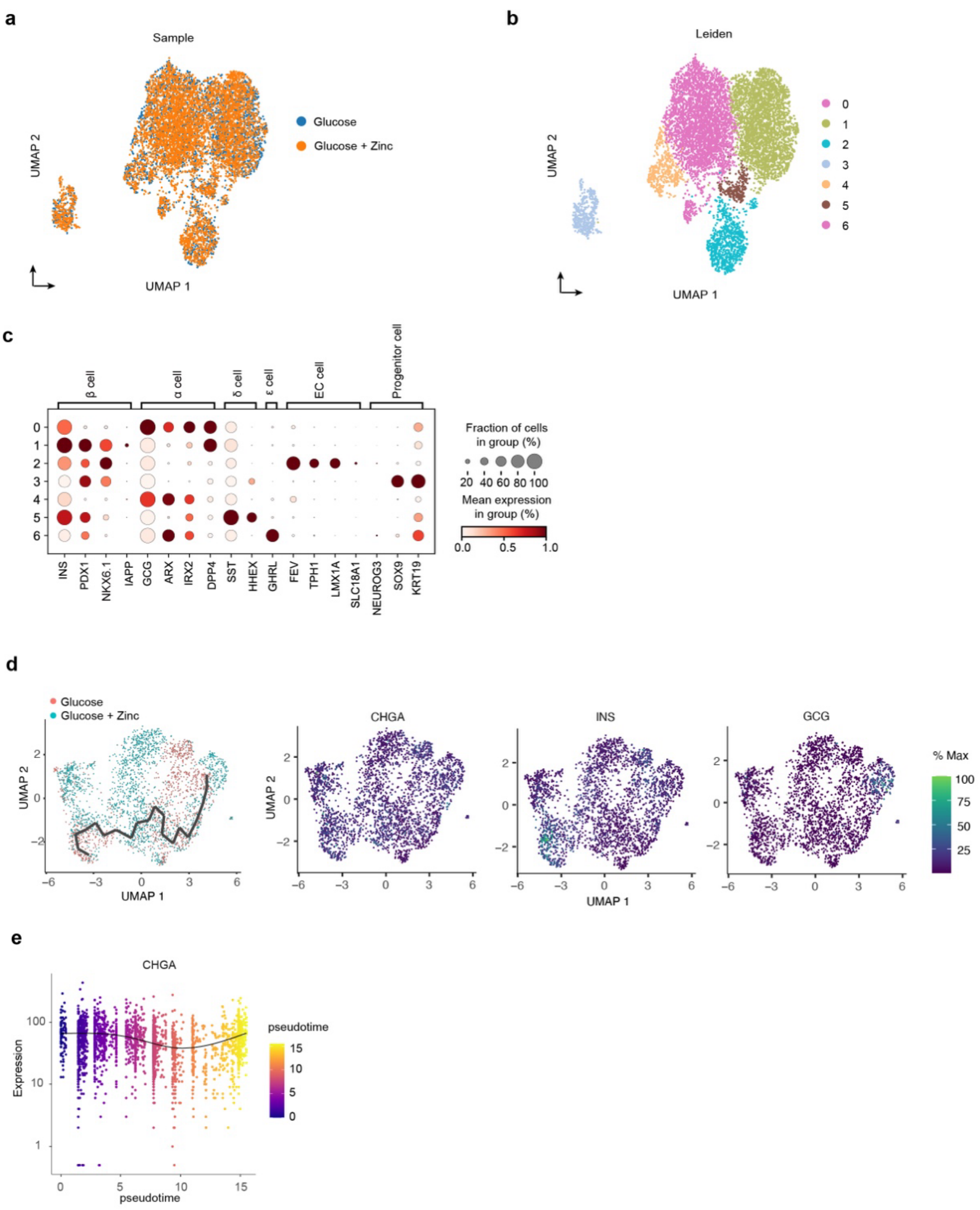

Supplementary information, Figure S14 Single-cell analysis of SC-islets. a UMAP plot showing the distribution of cells from two groups: glucose (blue) and glucose with excessive zinc (orange). **b** UMAP plot of pancreatic cells colored by Leiden clusters. **c** Dot plot showing the mean expression levels and fraction of cells expressing key marker genes across different Leiden clusters. **d** UMAP plots along pseudotime showing cell distribution under glucose and glucose with excessive zinc. Feature plots display expression of *CHGA*, *INS*, and *GCG* along pseudotime. **e** Pseudotime analysis of *CHGA* expression.
