# Supplementary material for: Zinc accumulation-induced integrated stress response triggers β-cell identity loss
Source: Cell Res. 2026 Jan 28;36(5):359–76. doi: 10.1038/s41422-026-01222-y (PMC13092640; doi:10.1038/s41422-026-01222-y)
Supplement: Supplementary file 15 — Supplementary information, Figure 15 [file 41422_2026_1222_MOESM15_ESM.pdf]

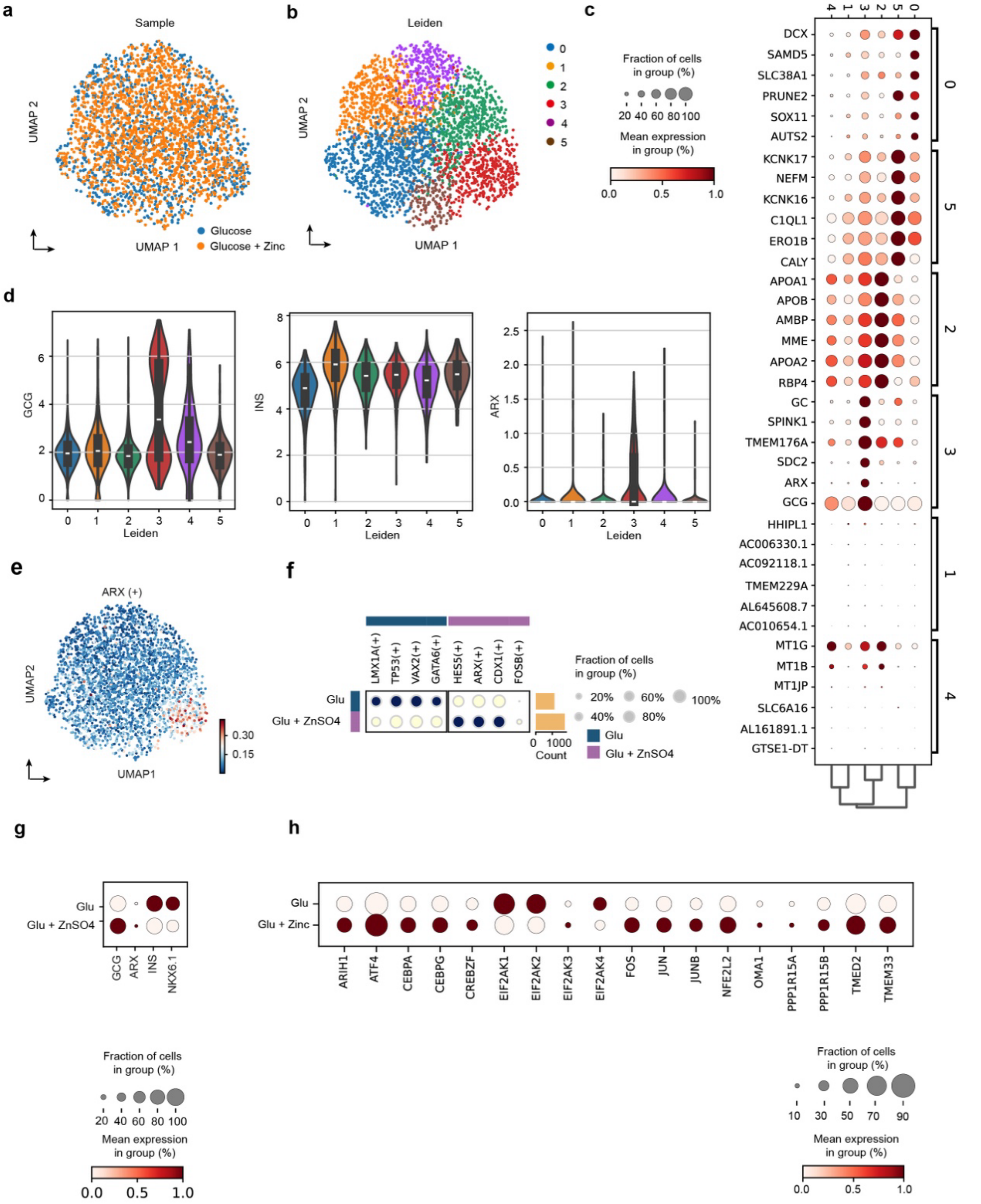


**Supplementary information, Figure S15 Single-cell analysis of β cell subclusters with excessive zinc treatment in SC-islets. a** UMAP plot showing the distribution of β cells from glucose (blue) and glucose with excessive zinc (orange). **b** UMAP plot labeled with Leiden clusters, indicating distinct β cell subclusters. **c** Dot plot showing the mean expression levels and fraction of cells expressing key marker genes across different Leiden clusters. **d** Violin plots showing the expression levels of *GCG*, *INS* and *ARX* in β cell subclusters defined by Leiden clusters. **e** SCENIC-based transcription factor activity analysis reveals the activation of ARX in subcluster 3. **f** Dot plot shows upregulation of ARX transcriptional activity in β cells of the excessive zinc treatment group. **g** Dot plot demonstrates increased expression of GCG and ARX, along with decreased expression of INS and NKX6.1 in β cells of the

excessive zinc treatment group. **h** Dot plot showing the mean expression levels and fraction of cells expressing ISR marker genes across different Leiden clusters with or without excessive zinc treatment under high glucose treatment.
