# Supplementary material for: Zinc accumulation-induced integrated stress response triggers β-cell identity loss
Source: Cell Res. 2026 Jan 28;36(5):359–76. doi: 10.1038/s41422-026-01222-y (PMC13092640; doi:10.1038/s41422-026-01222-y)
Supplement: Supplementary file 16 — Supplementary information, Figure 16 [file 41422_2026_1222_MOESM16_ESM.pdf]

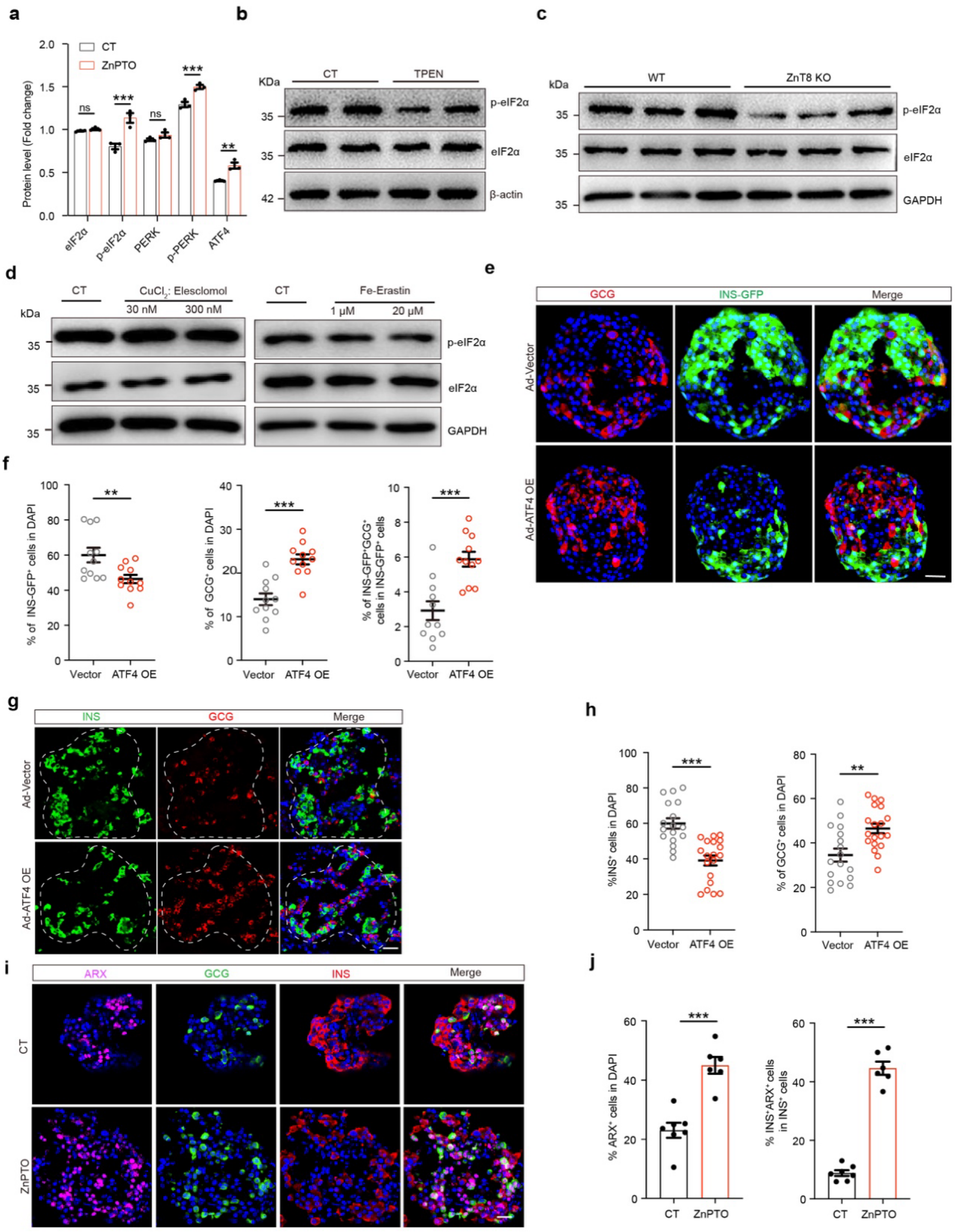

Supplementary information, Figure S16 Excessive Zinc-induced β cell identity loss is mediated by ISR and subsequent ATF4 activation. a Quantification for expression changes of eIF2α, phosphorylated-eIF2α, PERK, phosphorylated-PERK and ATF4 in SC-islets treated with or without ZnPTO. n = 3. **b** Western blot analysis for expression changes of phosphorylated eIF2α in SC-islets with or without 0.5 μM TPEN treatment. **c** Western blot analysis for expression changes of phosphorylated eIF2α in WT and ZnT8 KO SC-islets. **d** Western blot analysis for expression changes of phosphorylated eIF2α in SC-islets treated with CuCl2: Elesclomol (copper ionophore) for 72 h; Fe Erastin (reagent enriching intracellular iron level) for 24h. **e, f** Representative immunofluorescent images (**e**) and

quantification (**f**) for percentages of INS-GFP$^+$ cells and GCG$^+$ cells among the total number of DAPI$^+$ cells, as well as proportion of bi-hormonal INS-GFP$^+$GCG$^+$ cells among total INS-GFP$^+$ cells in SC-islets infected with adenovirus carrying either an empty vector or ATF4 OE construct. n = 11. Scale bar, 50 μm. **g, h** Representative immunofluorescent images (**g**) and quantification (**h**) for percentages of INS$^+$ cells (green) and GCG$^+$ cells (red) among the total number of DAPI$^+$ cells (blue) in human primary islets infected with adenovirus carrying either an empty vector (n = 17) or ATF4 OE (n = 19) construct. Scale bar, 25 μm. **i, j** Representative immunofluorescent images (**i**) and quantification (**j**) for percentages of ARX$^+$ cells (magenta) among the total number of DAPI$^+$ cells (blue) and the proportion of INS$^+$ARX$^+$ cells among total INS$^+$ cells in human primary islets with (n = 6) or without (n = 7) ZnPTO treatment. Unpaired two-tailed *t* test was used to analyze in this figure. *$p < 0.05$, **$p < 0.01$, ***$p < 0.001$. Data are presented as mean ± s.e.m. Individual data points are shown for all bar graphs.
