# Supplementary material for: Zinc accumulation-induced integrated stress response triggers β-cell identity loss
Source: Cell Res. 2026 Jan 28;36(5):359–76. doi: 10.1038/s41422-026-01222-y (PMC13092640; doi:10.1038/s41422-026-01222-y)
Supplement: Supplementary file 17 — Supplementary information, Figure 17 [file 41422_2026_1222_MOESM17_ESM.pdf]

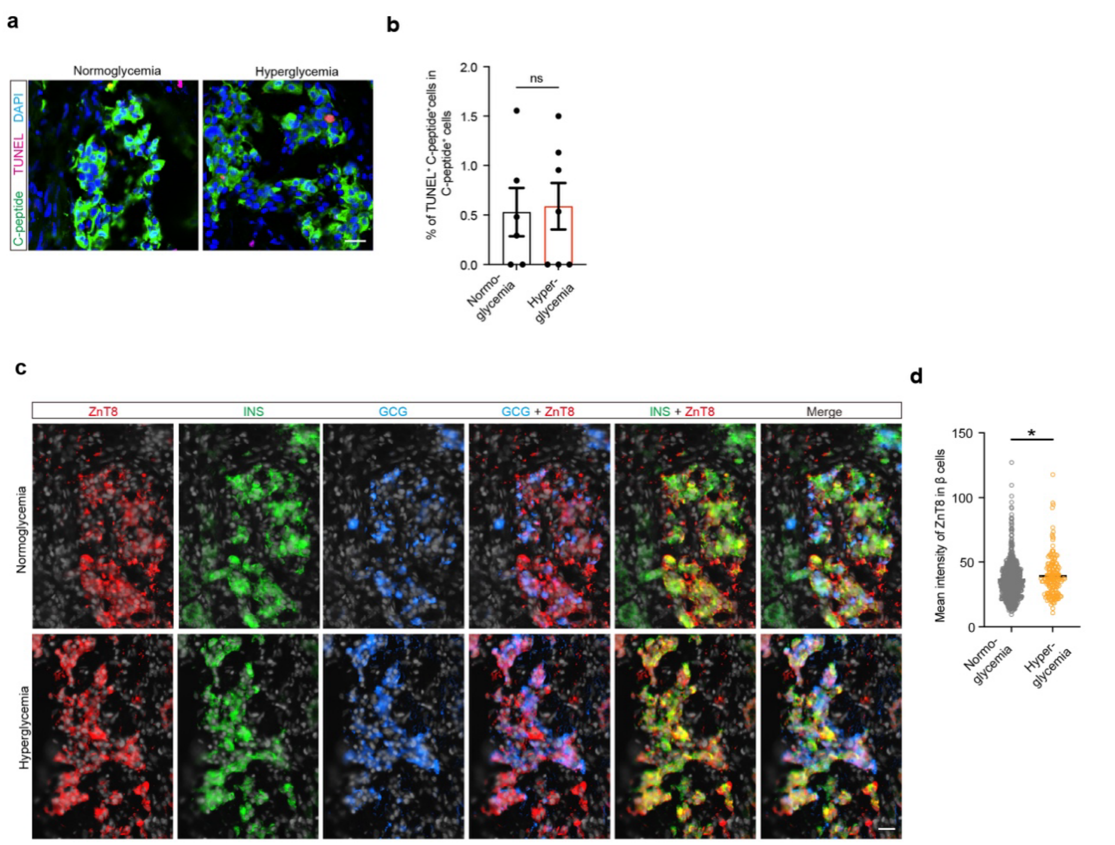

Supplementary information, Figure S17 Additional analysis of hyperglycemia induces human primary β cell identity loss. a, b Representative immunofluorescent images (**a**) and the quantification (**b**) for percentages of TUNEL$^+$C-peptide$^+$ cells in total C-peptide$^+$ cells from human primary β cells implanted in normoglycemic mice (n = 6) or hyperglycemic mice (n = 7). Scale bar, 25 μm. **c**, **d** Representative immunofluorescent images (**c**) and the quantification (**d**) for mean intensity of ZnT8 in human primary β cells implanted in normoglycemic (n = 674) or hyperglycemic mice (n = 156). Scale bar, 25 μm. Unpaired two-tailed *t* test was used to analyze in this figure. *$p < 0.05$, **$p < 0.01$, ***$p < 0.001$, ns, no significance. Data are presented as mean ± s.e.m. Individual data points are shown for all bar graphs.
