# Supplementary material for: Zinc accumulation-induced integrated stress response triggers β-cell identity loss
Source: Cell Res. 2026 Jan 28;36(5):359–76. doi: 10.1038/s41422-026-01222-y (PMC13092640; doi:10.1038/s41422-026-01222-y)
Supplement: Supplementary file 18 — Supplementary information, Figure 18 [file 41422_2026_1222_MOESM18_ESM.pdf]

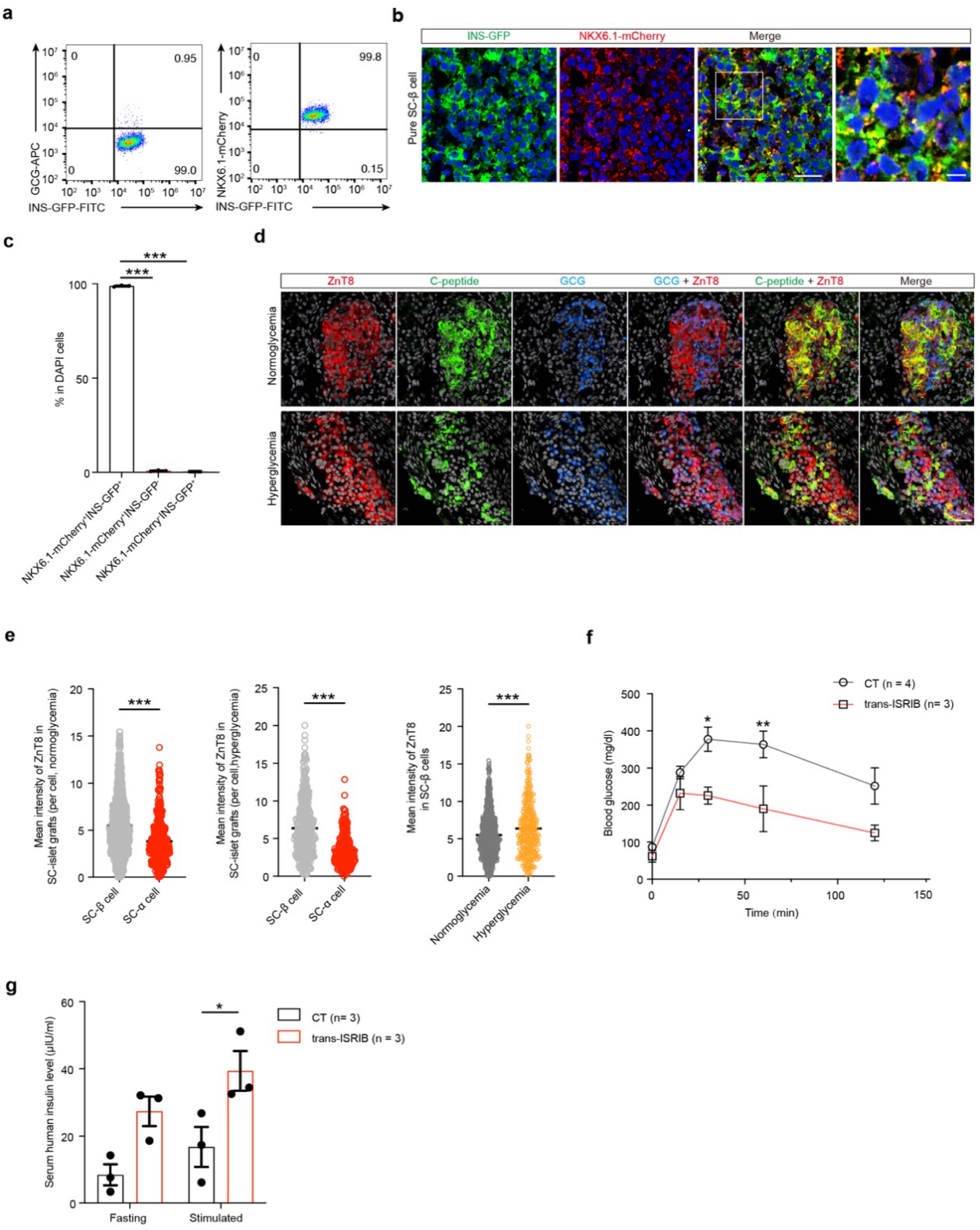

Supplementary information, Figure S18 Additional analysis of hyperglycemia induces SC-β cell identity loss. a Representative FACS plots for the percentages of INS-GFP$^+$GCG$^-$cells and INS-GFP$^+$NKX6.1-mCherry$^+$ cells in pure SC-β cell clusters. **b, c** Representative fluorescent images (**b)** and the quantification (**c**) for the percentages of NKX6.1-mCherry$^+$INS-GFP$^+$, NKX6.1-mCherry$^+$INS-GFP$^-$ and NKX6.1-mCherry$^-$INS-GFP$^+$ among the total number of DAPI$^+$ cells in adherent pura SC-β cells. n = 3. Scale bar in high magnification, 5 μm; Scale bar in low magnification, 25 μm. **d**, **e** Representative immunofluorescent images (**d**) and the quantification (**e**) for mean intensity of ZnT8 in SC-α cells and SC-β cells implanted in normoglycemic (SC-β cells, n = 1239; SC-α cells, n = 464) or hyperglycemic mice (SC-β cells, n = 428; SC-α cells, n = 224). Scale bar, 25 μm. **f** i.p. GTT in the diabetic mice

transplanted with SC-islets and administrated with or without trans-ISRIB. n = 3. **g** GSIS assay measuring serum human insulin levels of the diabetic mice transplanted with SC-islets and administrated with(n = 3) or without trans-ISRIB (n = 4). One-way ANOVA with Dunnett's was used to analyze for **c**. Unpaired two-tailed *t* test was used to analyze for **e**. Two-way ANOVA with Sidak's multiple-comparisons was used to analyze for **f**. Two-way ANOVA with Tukey's multiple-comparisons was used to analyze for **g**. *$p < 0.05$, **$p < 0.01$, ***$p < 0.001$. Data are presented as mean ± s.e.m. Individual data points are shown for all bar graphs.
