# Supplementary material for: Zinc accumulation-induced integrated stress response triggers β-cell identity loss
Source: Cell Res. 2026 Jan 28;36(5):359–76. doi: 10.1038/s41422-026-01222-y (PMC13092640; doi:10.1038/s41422-026-01222-y)
Supplement: Supplementary file 19 — Supplementary information, Figure 19 [file 41422_2026_1222_MOESM19_ESM.pdf]

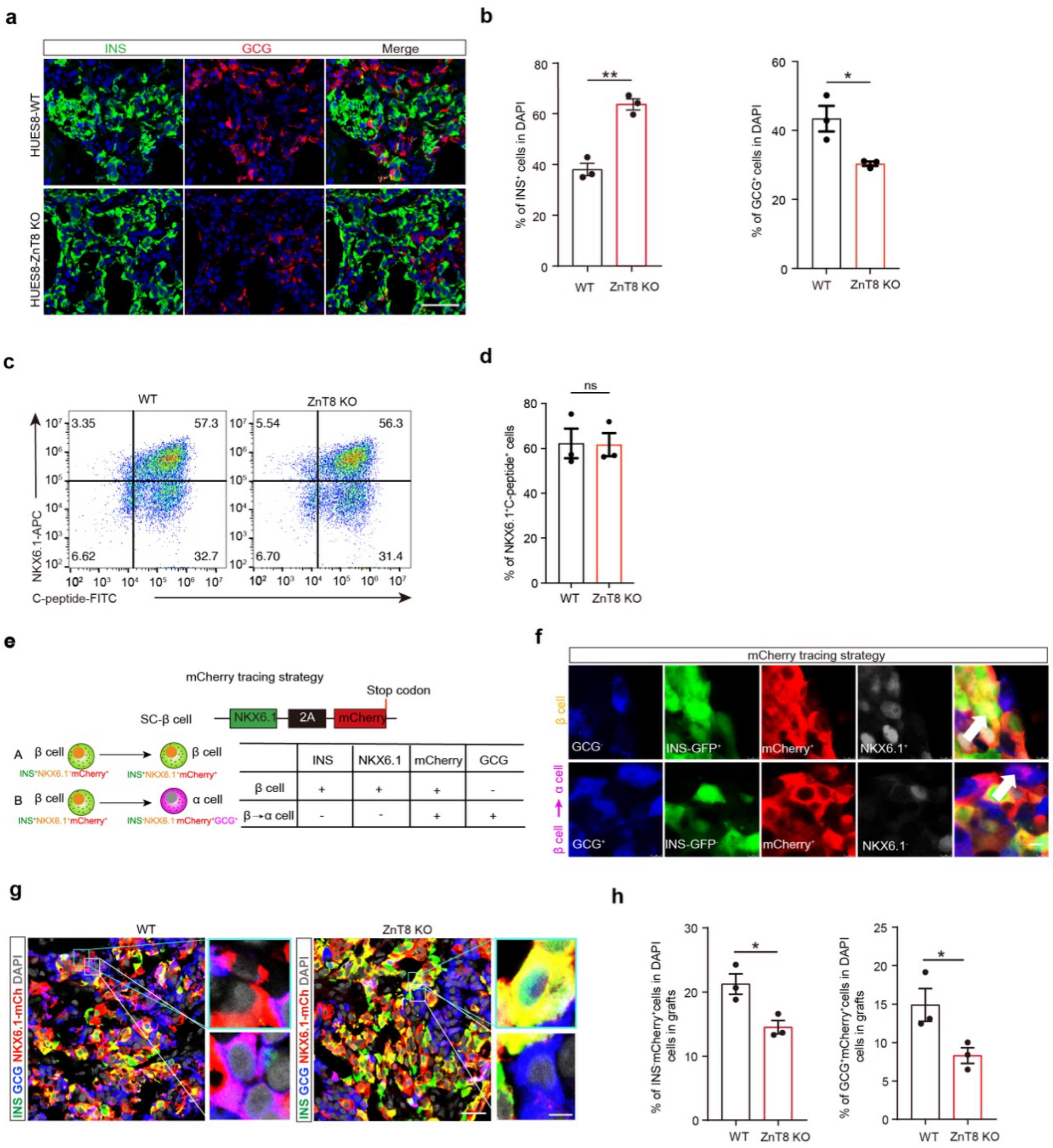

Supplementary information, Figure S19 Additional analysis of ZnT8 deficiency protects SC-β cell identity loss. a, b Representative immunofluorescent images **(a)** and quantification **(b)** for percentages of SC-β cells (INS, green) and SC-α cells (GCG, red) among the total number of DAPI^{+} cells (blue) in WT and ZnT8 KO SC-islet grafts transplanted into hyperglycemic mice. n = 3. Scale bar, 50 μm. **c, d** Representative FACS plots (**c**) and quantification (**d**) of percentages of NKX6.1^{+}C-peptide^{+} cells in WT and ZnT8 KO SC-islets. n = 3. **e, f** Schematic (**e**) and representative immunofluorescent images (**f**) of the mCherry tracing strategy in SC-β cell. Scale bar, 5 μm. **g, h** Representative immunofluorescent mCherry tracing images (**g**) and quantification (**h**) of WT and KO SC-islet grafts stained with INS (green), GCG (blue) and NKX6.1-mCherry (red). n = 3. Scale bar in high magnification, 5 μm; Scale bar in low magnification, 25 μm. Unpaired two-tailed *t* test was used to analyze in this figure. *$p < 0.05$, **$p < 0.01$, ***$p < 0.001$, ns, no significance. Data are presented as mean ± s.e.m. Individual data points are shown for all bar graphs.
