# Supplementary material for: Zinc accumulation-induced integrated stress response triggers β-cell identity loss
Source: Cell Res. 2026 Jan 28;36(5):359–76. doi: 10.1038/s41422-026-01222-y (PMC13092640; doi:10.1038/s41422-026-01222-y)
Supplement: Supplementary file 20 — Supplementary information, Figure 20 [file 41422_2026_1222_MOESM20_ESM.pdf]

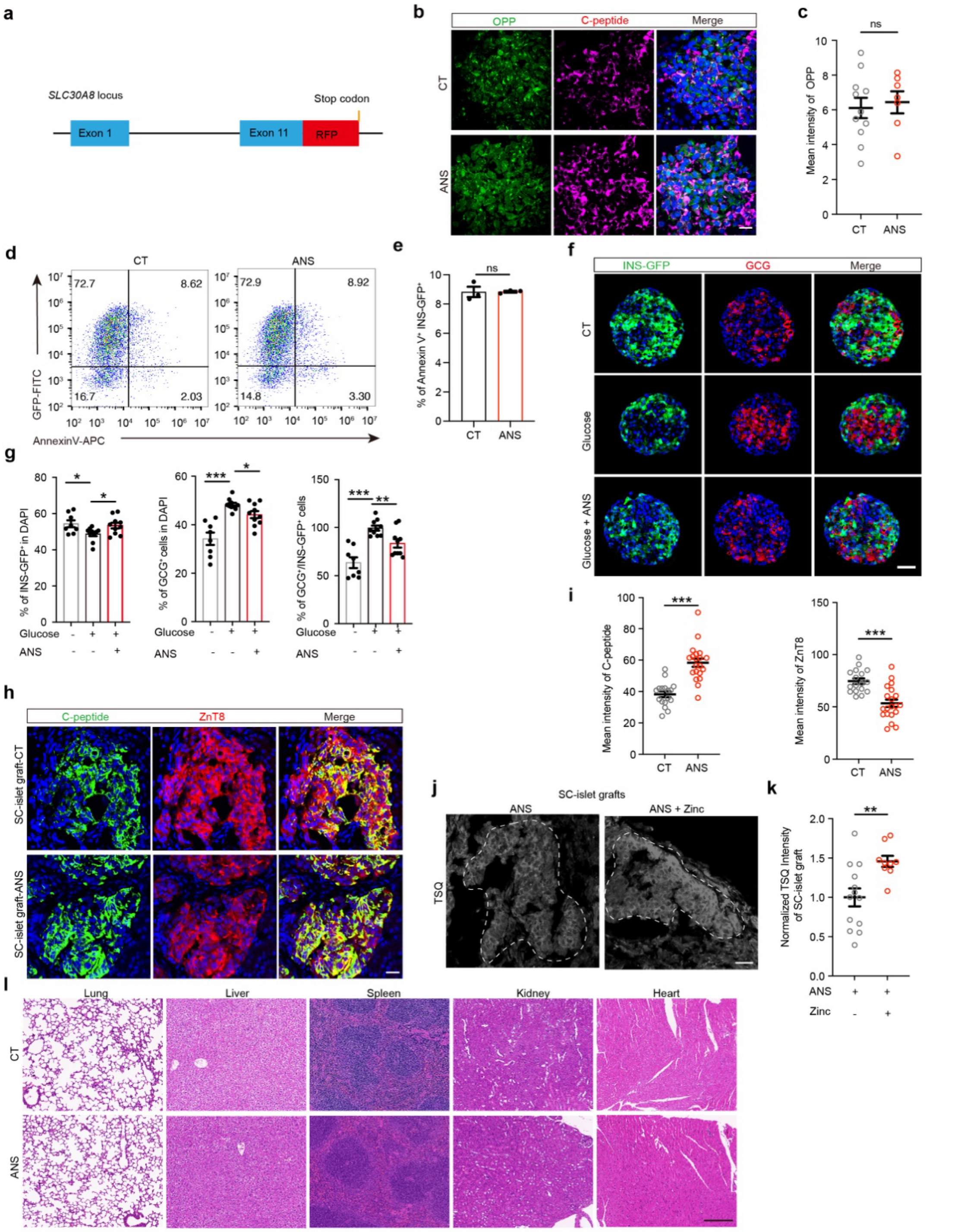

Supplementary information, Figure S20 ANS protects SC-β cell identity under high glucose. **a** Schematic diagram illustrating the construction strategy of ZnT8-RFP in the MEL1-*INS*$^{WT/GFP}$ cell line. **b, c** Representative immunofluorescent images (**b**) and mean intensity measurements (**c**) for mean intensity of OPP in adherent SC-islets with (n = 7) or without 25 nM ANS treatment (n = 11). Scale bar, 10 μm. **d, e** Representative FACS plots (**d**) and quantification (**e**) of Annexin V^{+}INS-GFP^{+} populations in SC-islets treated with ANS. n = 3. **f, g** Representative immunofluorescent images (**f**) and the quantification (**g**) for percentages of INS-GFP^{+} cells (green) and GCG^{+} cells (red) among the total number of DAPI^{+} cells, as well as the ratio of GCG^{+} cells to INS-GFP^{+} cells in the SC-islet treated with

control (n = 8), high glucose (n = 10) and high glucose with ANS (n = 10). Scale bar, 50 µm. **h, i** Representative immunofluorescent images (**h**) and the mean intensity measurements (**i**) for C-peptide (green) and ZnT8 (red) in the SC-islets grafts from hyperglycemic mice treated with (n = 20) or without (n = 17) ANS. Scale bar, 25 µm. **j, k** TSQ staining (**j)** and normalized mean intensity measurements (**k**) for the SC-islet engrafts from hyperglycemic mice treated with (n = 9) or without (n = 13) zinc supplementation under ANS injection. Scale bar, 25 µm. **l** Representative H&E staining images of major organs from the mice administrated with or without ANS. Scale bar, 200 µm. Unpaired two-tailed *t* test was used to analyze in this figure. *$p < 0.05$, **$p < 0.01$, ***$p < 0.001$, ns, no significance. Data are presented as mean ± s.e.m. Individual data points are shown for all bar graphs.
