# Supplementary material for: Zinc accumulation-induced integrated stress response triggers β-cell identity loss
Source: Cell Res. 2026 Jan 28;36(5):359–76. doi: 10.1038/s41422-026-01222-y (PMC13092640; doi:10.1038/s41422-026-01222-y)
Supplement: Supplementary file 21 — Supplementary information, Table S1 [file 41422_2026_1222_MOESM21_ESM.pdf]

Supplementary information, Table S1 Clinical data of ND and patients with T2D from PANC-DB.

Donor ID	Age	BMI	HbA1c (%)	Clinical diagnosis	Disease duration
HPAP-022	39	34.7	4.7	ND	N/A
HPAP-026	24	20.8	4.9	ND	N/A
HPAP-027	31	32.71	4.4	ND	N/A
HPAP-034	13	18.6	5.2	ND	N/A
HPAP-035	35	26.91	5.2	ND	N/A
HPAP-036	23	16	5.2	ND	N/A
HPAP-037	35	21.9	5.3	ND	N/A
HPAP-039	5	16.3	6.8	ND	N/A
HPAP-040	35	23.98	5.4	ND	N/A
HPAP-042	1	17.9	5.6	ND	N/A
HPAP-044	3	12	5.3	ND	N/A
HPAP-051	43	45.49	6.7	T2D	5-6 years
HPAP-052	27	38.72	5.2	ND	N/A
HPAP-053	58	24.2	5.6	ND	N/A
HPAP-054	40	30.85	4.8	ND	N/A
HPAP-056	33	32.89	5.6	ND	N/A
HPAP-058	34	29.26	9.4	T2D	<1 year
HPAP-059	35	37.96	5.1	ND	N/A
HPAP-065	40	37.47	9.5	T2D	3 years
HPAP-069	47	24.86	6.7	ND	N/A
HPAP-070	55	17.09	7	T2D	16 years
HPAP-077	47	32.78	5.7	ND	N/A
HPAP-079	52	28.39	6.8	T2D	10 years
HPAP-080	22	35.71	5.4	ND	N/A
HPAP-081	45	28.91	7.9	T2D	20 years
HPAP-082	25	23.96	5.6	ND	N/A
HPAP-083	45	35.62	5	T2D	2-3 years
HPAP-085	48	42.27	7.3	T2D	6-10 years
HPAP-088	37	32.82	10.3	T2D	<1 month
HPAP-090	59	32.32	8.7	T2D	>3 years
HPAP-091	50	35.58	6.9	T2D	3 months
HPAP-093	50	34.98	4.9	ND	N/A
HPAP-099	28	24.7	5	ND	N/A
HPAP-100	41	28.83	10.7	T2D	7-8 years
HPAP-101	55	38.07	5	T2D	NA
HPAP-104	4	20.63	4.9	ND	NA
HPAP-105	51	28.1	5.2	T2D	NA
HPAP-109	59	29.49	7.5	T2D	<5 years
