# Supplementary material for: Zinc accumulation-induced integrated stress response triggers β-cell identity loss
Source: Cell Res. 2026 Jan 28;36(5):359–76. doi: 10.1038/s41422-026-01222-y (PMC13092640; doi:10.1038/s41422-026-01222-y)
Supplement: Supplementary file 22 — Supplementary information, Table S2 [file 41422_2026_1222_MOESM22_ESM.pdf]

Supplementary information, Table S2 Clinical data of ND and patients with T2D of Fig. 1d, e; Fig. 1l, m; Supplementary information, Fig. S6f.

Donor ID	Age	BMI	HbA1c (%)	Clinical diagnosis
1	67	19.38	5.3	ND
2	76	24.39	6.5	ND
3	67	28.04	5.4	ND
4	77	28.76	9.5	T2D
5	75	24.44	9.1	T2D
6	67	25.35	8	T2D
