# Supplementary material for: Zinc accumulation-induced integrated stress response triggers β-cell identity loss
Source: Cell Res. 2026 Jan 28;36(5):359–76. doi: 10.1038/s41422-026-01222-y (PMC13092640; doi:10.1038/s41422-026-01222-y)
Supplement: Supplementary file 23 — Supplementary information, Table S3 [file 41422_2026_1222_MOESM23_ESM.pdf]

Supplementary information, Table S3 Clinical data of ND and patients with T2D of Fig. 1f, g.

Donor ID	Age	BMI	Blood glucose	Clinical diagnosis
2303160	67	23.62	12.2	T2D
2304153	67	21.34	11.6	T2D
2304403	53	24.1	20.4	T2D
2304708	76	31.11	12.8	T2D
2304961	61	23.38	7.6	T2D
2305226	73	25.65	13.1	T2D
2306072	78	28.72	13	T2D
2307958	65	23.12	13.5	T2D
2309638	60	26.56	10.2	T2D
2310249	61	19.53	14.9	T2D
2404934	76	23	5.2	ND
2404936	53	29	6.7	ND
2404937	58	26	5.2	ND
2405209	54	19	6.6	ND
2405792	73	28	8	ND
2405794	70	26	5.3	ND
2405795	63	25	4.4	ND
2405796	70	22	5.8	ND
2406371	56	29	6.2	ND
2406372	79	21	5.3	ND
