# Supplementary material for: Zinc accumulation-induced integrated stress response triggers β-cell identity loss
Source: Cell Res. 2026 Jan 28;36(5):359–76. doi: 10.1038/s41422-026-01222-y (PMC13092640; doi:10.1038/s41422-026-01222-y)
Supplement: Supplementary file 24 — Supplementary information, Table S4 [file 41422_2026_1222_MOESM24_ESM.pdf]

Supplementary information, Table S4 Comparison of serum biochemistry and complete blood count between HFD-Fed mice with or without zinc supplementation

Indexes	HFD	HFD+Zn	*p*-value
Serum copper (μmol/L)	15.34±2.621	13.34±2.033	0.5784
Serum iron (μmol/L)	19.29±5.676	27.66±2.602	0.2512
Serum magnesium (mmol/L)	0.9300±0.091	0.8167±0.003	0.2800
Serum calcium (mmol/L)	2.637±0.023	2.587±0.024	0.2098
ALT (U/L)	186.8±28.23	175.6±66.36	0.8847
AST (U/L)	231.1±15.1	198.5±34.25	0.4329
UREA (mmol/L)	15±9.629	7.5±1.59	0.4852
CREA (μmol/L)	19.49±10.93	7.687±0.4437	0.3414
CK (U/L)	918.7±410.8	713.8±77.49	0.6497
WBC (10^3/uL)	10.87±1.549	9.777±1.970	0.6852
NEUT# (10^3/uL)	1.017±0.096	1.26±0.292	0.4730
LYMPH# (10^3/uL)	9.177±1.460	7.88±1.696	0.5934
MONO# (10^3/uL)	0.38±0.055	0.53±0.134	0.3605
EO# (10^3/uL)	0.287±0.085	0.097±0.028	0.1017
