# Supplementary material for: Zinc accumulation-induced integrated stress response triggers β-cell identity loss
Source: Cell Res. 2026 Jan 28;36(5):359–76. doi: 10.1038/s41422-026-01222-y (PMC13092640; doi:10.1038/s41422-026-01222-y)
Supplement: Supplementary file 25 — Supplementary information, Table S5 [file 41422_2026_1222_MOESM25_ESM.pdf]

Supplementary information, Table S5 Comparison of serum biochemistry between control and ANS administered

**Indexes**	**CT**	**ANS**	***p*-value**
ALT(U/L)	32±2.273	33.5±2.533	0.6748
AST(U/L)	93.25±11.16	121.8±9.34	0.0979
CK(U/L)	983.5±174.4	1351±115.1	0.1290
CRE(μmol/L)	14.17±1.064	17.44±1.202	0.0878
LDH(U/L)	384.6±90.12	461.1±48.28	0.4826
