# Supplementary material for: Zinc accumulation-induced integrated stress response triggers β-cell identity loss
Source: Cell Res. 2026 Jan 28;36(5):359–76. doi: 10.1038/s41422-026-01222-y (PMC13092640; doi:10.1038/s41422-026-01222-y)
Supplement: Supplementary file 26 — Supplementary information, Table S6 [file 41422_2026_1222_MOESM26_ESM.pdf]

Supplementary information, Table S6 Differentiation protocol of SC-islet

Day	Stage	basal medium	Factor	Final Concentration
Day 0	**Stage 1** (Definitive Endoderm)	RPMI1640	Activin A	100 ng/ml
			Chir99021	2 μM
Day 1-2			BMP4	0.25 ng/ml
			bFGF	5 ng/ml
			Activin A	100 ng/ml
			vEGF	10 ng/ml
			Ascorbic acid	50 μg/ml
Day 3-5	**Stage 2** (Primitive Gut Tube)	SFD	FGF10	50 ng/ml
			Wnt3a	3 ng/ml
			Ascorbic acid	50 μg/ml
Day 6-7	**Stage 3** (Posterior Foregut)	DMEM	Ascorbic acid	50 μg/ml
			SANT1	0.25 μM
			Retinoic acid	2 μM
			NOGGIN	50 ng/ml
			FGF10	50 ng/ml
Day 8-10	**Stage 4** (Pancreatic Progenitors)	DMEM	Ascorbic acid	50 μg/ml
			NOGGIN	50 ng/ml
			hEGF	50 ng/ml
			Nicotinamide	10 mM
Day 11-13	**Stage 5** (Endocrine Progenitors)	MCDB131	SANT1	0.25 μM
			Alk5i-II	10 μM
			LDN193189	100 nM
			Retinoic acid	0.05 μM
			hEGF	50 ng/ml
			Triiodothyronine	1 μM
Day 14-19	**Stage 6** (Immature SC-β Cell)	MCDB131	Alk5i-II	10 μM
			LDN193189	100 nM
			Gamma secretase inhibitor XX	100 nM
			hEGF	50 ng/ml
			Triiodothyronine	1 μM
Day 20-33	**Stage 7** (Mature SC-β Cell)	MCDB131	Triiodothyronine	1 μM
			Alk5i-II	10 μM
			Trolox	10 μM
			R428	2 μM
			N-Cys	1 mM
