# Supplementary material for: Zinc accumulation-induced integrated stress response triggers β-cell identity loss
Source: Cell Res. 2026 Jan 28;36(5):359–76. doi: 10.1038/s41422-026-01222-y (PMC13092640; doi:10.1038/s41422-026-01222-y)
Supplement: Supplementary file 27 — Supplementary information, Table S7 [file 41422_2026_1222_MOESM27_ESM.pdf]

Supplementary information, Table S7 Primers used for real-time PCR

Gene Name	Forward Primer Sequence	Reverse Primer Sequence
HRI	GCCTTGGAAGCACAAACTTCA	GTAGGACCTTCATGCAAACTGTT
PKR	TGGAAAGCGAACAAGGAGTAAG	CCAAAGCGTAGAGGTCCACTT
PERK	GTCCGGAACCAGACGATGAG	GGCTGGATGACACCAAGGAA
GCN2	TCTTTGAACTGGCTTACCACG	GCAGGATTTCACGTTGCTCC
ATF4	GACCGAAATGAGCTTCCTGA	ACCCATGAGGTTTGAAGTGC
ATF6	TCCTCGGTCAGTGGACTCTTA	CTTGGGCTGAATTGAAGGTTTTG
IRE1α	CACAGTGACGCTTCCTGAAAC	GCCATCATTAGGATCTGGGAGA
XBP1	CCTGGTTGCTGAAGAGGAGG	CCATGGGGAGATGTTCTGGAG
GRP78	CACAGTGGTGCCTACCAAGA	TGATTGTCTTTTGTCAGGGGT
SLC30A8	GAGCGGAGAACGGTGATTT	GAAGGTGGGAACGGAGAAC
